# Integrated Metabolomics and Transcriptomics Analysis of Exogenous Arginine-Mediated Sucrose Accumulation in Sugarcane

**DOI:** 10.3390/ijms27125476

**Published:** 2026-06-17

**Authors:** Hong-Bo Liu, Tanweer Kumar, Xiu-Qin Lin, Chao-Hua Xu, Jun Mao, Chun-Yan Kong, Xu-Juan Li, Chun-Yan Tian, Wajid Khan, Nur-ul-Haq, Li Yao, Pei-Fang Zhao, Jia-Yong Liu, Jun-Gang Wang, Xin Lu

**Affiliations:** 1 State Key Laboratory of Tropical Crop Breeding, Sugarcane Research Institute, Yunnan Academy of Agricultural Sciences, Yunnan Key Laboratory of Sugarcane Genetic Improvement, Kaiyuan 661699, China; 2 Sugar Crops Research Institute (SCRI), Agriculture Research Department, Charsadda Road, Mardan 23210, Khyber Pakhtunkhwa, Pakistan; 3 State Key Laboratory of Tropical Crop Breeding, Institute of Tropical Bioscience and Biotechnology, Chinese Academy of Tropical Agricultural Sciences, Haikou 571101, China; 4 Key Laboratory of Biology and Genetic Resources of Tropical Crops, Sanya Research Institute, Chinese Academy of Tropical Agricultural Sciences, Sanya 572024, China

**Keywords:** sugarcane, arginine, metabolomics, transcriptomics, sucrose accumulation, multi-omics integration

## Abstract

The improvement of sucrose yield in sugarcane is impeded by the crop’s complex polyploid genome and slow progress in breeding. To clarify how arginine (Arg) regulates sugar metabolism and identify key genes associated with sucrose transport and accumulation in sugarcane, a screening experiment was performed by spraying L-arginine hydrochloride on the leaves and leaf sheaths of three sugarcane varieties (YZ05-51, YZ08-1609, and YT93-159), which differ in growth vigor, leaf morphology and other phenotypic traits. YZ05-51 exhibited the most prominent sugar-increasing effect, and subsequent optimization experiments on its leaf sheaths revealed that 20 g/mu L-arginine hydrochloride at pH 7.0 was optimal, significantly enhancing stem sucrose content. Transcriptomic analysis revealed the upregulation of genes related to sucrose synthesis and transport, with candidate genes enriched in pathways such as starch-sucrose metabolism, glycolysis/gluconeogenesis, and ATP-binding cassette (ABC) transporters. Metabolomic analysis detected 32 sugar metabolites across three categories, of which 24 were differentially abundant (e.g., glucose, galactose, fructose, and mannose). Integrated multi-omics analysis identified key regulatory genes, including *SBEs* and *TPS1* (sucrose synthesis and carbon flux regulation), *RBSK*, *α-amylases*, *GH28* (starch breakdown, glycolysis, and sugar mobilization), ABC transporters, *GTs*, and *TIM10*/*TIM12* (sucrose transporter). Collectively, these analyses demonstrate enhanced activity of genes and metabolites involved in sucrose synthesis/transport in leaf sheaths, accompanied by reduced synthesis of other monosaccharides and oligosaccharides. Vigorously metabolizing leaf sheaths is more conducive to sucrose transport. This study provides valuable insights into the molecular mechanisms underlying Arg-mediated sucrose accumulation specifically in the sugarcane YZ05-51 sugarcane, highlighting its critical regulatory roles.

## 1. Introduction

Sugarcane (*Saccharum* spp.) is a semi-perennial plant with high photosynthetic efficiency. It is widely grown as an agronomic and industrial crop for sugar and bioethanol production [1,2], and serves as an optimal green energy crop for addressing global environmental pollution issues. However, enhancing energy capture in sugarcane and boosting sucrose yield remain unresolved challenges. Due to the complex nature of the sugarcane genome, high ploidy, narrow gene pool, and long breeding cycle, improving sucrose content remains challenging. The synthesis and accumulation of sucrose in sugarcane is a highly intricate process that involves multiple interactions at various levels of cellular organization. Extensive research has been conducted to elucidate the mechanisms underlying sucrose accumulation in sugarcane. Studies have explored transcriptomic and metabolomic approaches [3,4], genes involved in sucrose metabolism and transport [5,6], gene localization and genomic mapping [7], and the identification of quantitative trait loci (QTL) associated with sucrose content [8,9,10]. In addition, studies have also explored genetic variations in haplotypes and alleles differing in sucrose accumulation capacity [11].

However, chemical crop regulation has emerged as an advanced and efficient technology in the agricultural sector, utilizing plant growth regulators to regulate crop growth, development, and various biochemical and physiological processes [12]. Arg serves as a plant growth regulator with a high nitrogen-to-carbon ratio and plays a crucial role as a key component of the catalytic site in specific enzymes, highlighting its versatility as an amino acid [13,14]. It functions as a precursor in the synthesis of polyamines and signaling molecules, such as nitric oxide, and has been demonstrated to enhance plant resistance to biotic and abiotic stresses [14,15]. Therefore, arginine has great potential for application in sugarcane cultivation.

An integrated analysis of metabolomics and transcriptomics is crucial for identifying key traits, as it offers precise insights into metabolite accumulation and the regulatory genes involved. For instance, cane varieties are closely associated with sugar yield and yield-related parameters such as brix, sucrose, and fiber content [16]. A metabolomics approach was employed to analyze the leaves of various sugarcane varieties and quantify their metabolites and antioxidant activities. In addition, anthocyanin-related genes have been successfully identified through integrated metabolomic and transcriptomic analyses [17]. Metabolomic and proteomic studies have been effectively used to identify the functional proteins and genes associated with sucrose metabolism in sugarcane. Additionally, multi-omic correlation analysis was applied to explore the metabolic pathways and genes involved in carbon partitioning at different stages of culm development across various genotypes. These findings revealed a progressive increase in lignin, sucrose, carbon, and amino acid levels during culm maturation [4].

Despite extensive research on sucrose synthesis in leaves and sucrose content in culms, the characteristics of various sugarcane varieties remain inadequately understood. Furthermore, the role of leaf sheaths as significant tissues for sucrose transport has largely been ignored. We hypothesized that foliar L-arginine modulates the transport activity of the cell membrane and carbon-nitrogen signaling, potentially affecting sucrose allocation to culm tissues. In this study, we treated the leaves and leaf sheaths of three sugarcane varieties with different genotypes with different concentrations of arginine to explore the effects of arginine treatment on sugar metabolism in sugarcane. The YZ08-1609 variety is distinguished by its robust growth, broad leaves, dark green leaf sheaths, and thick culms. In contrast, the YZ05-51 variety exhibits purplish-red leaf sheaths during the early maturity stage, with rapid abscission of the middle leaf sheaths, while its leaves remain healthy and of medium width. Regarding the YT93-159 variety, its leaf sheaths retain a bright green color in the late growth stage but exhibit poor defoliation; its leaves exhibit premature senescence and severe mosaic virus infection, thereby decreasing photosynthetic efficiency. To verify the hypothesis regarding differences in sucrose accumulation among sugarcane varieties with Arg treatment, we applied exogenous L-arginine, which can improve plant nutritional status and cell membrane transport activity, and investigated the changes in sucrose content of the three varieties after spraying different concentrations of L-arginine hydrochloride. Subsequently, we selected the YZ05-51 variety with the most significant increase in sucrose content for transcriptomic and metabolomic analyses to clarify the mechanism by which arginine enhances sucrose content, to improve the identification and variety breeding of sugarcane germplasms with L-arginine-specific genotypes.

## 2. Results

### 2.1. Optimization of Sucrose Accumulation and Effects of Arginine and pH

**Figure 1:**
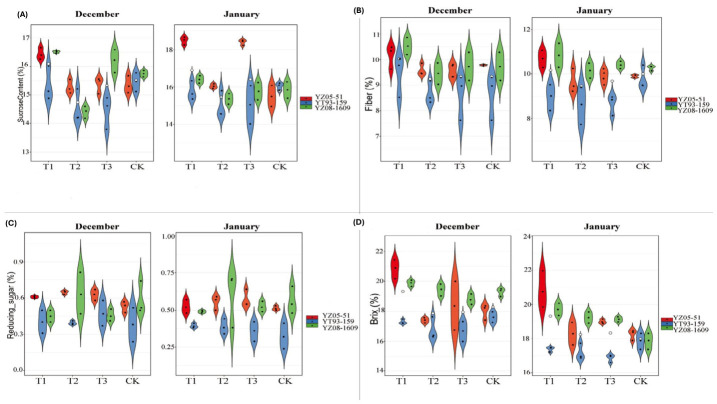
Distribution of sugarcane quality traits following early foliar arginine application in three major sugarcane-growing regions. Violin plots depict the full distribution of four critical quality metrics: (**A**) sucrose content %, (**B**) fiber content %, (**C**) reducing sugar content %, and (**D**) Brix %. Data represents two time-point assessments conducted in December and January post-arginine application, across three experimental arginine concentrations and T1 = 20 g/mu, T2 = 50 g/mu, T3 = 100 g/mu and an untreated control (CK). Three commercial sugarcane genotypes, YZ05-51, YT93-159, and YZ08-1609, were compared, each violin plot illustrating the density of biological replicates, internal points representing individual observations, and treatment effects visualized across harvest months.

**Figure 2:**
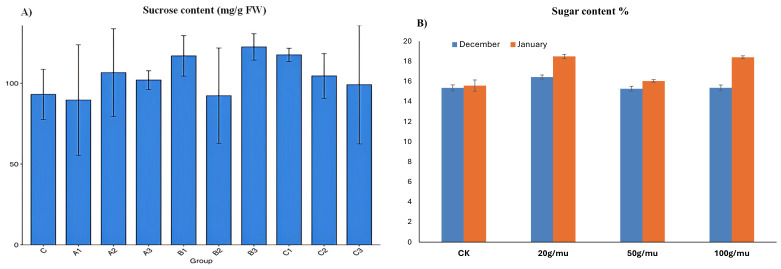
Effect of arginine on sucrose content in sugarcane cv. YZ05-51 sugarcane in three different months. (**A**) Effect of arginine on sucrose content in leaf sheaths at three concentrations of arginine compared with the control (C). 1, 2, and 3 represent 100, 50, and 20 g/mu of arginine, respectively, and letters A, B, and C represent the three pH levels (6.4, 7.0, and 7.4, respectively, in November). (**B**) Effect of optimum concentration of Arginine (20 g/mu at pH 7.0) compared with (CK) on sucrose content in the stem of YZ05-51 sugarcane in December and January.

Sucrose content measurements of the three sugarcane varieties in December 2022 and January 2023, following arginine treatment, revealed that Treatment 1 (20 g/mu) of YZ05-51 significantly increased brix and sucrose content across all three test sites when considering the three test sites as an integrated experimental unit, with a moderate increase in reducing sugar and a decrease in fiber content compared to the control group (Figure 1). The sucrose content in YZ05-51 treated with 50 g/mu (Treatment 2) and 100 g/mu (Treatment 3) showed no significant changes in December but exhibited slight increases in January. In contrast, YZ08-1609 showed less increase in sucrose content, whereas YT93-159 displayed a downward trend. These results indicate that low-concentration arginine specifically enhances sucrose accumulation in YZ05-51, consistent with the findings of [18]. Analysis of sucrose levels in YZ05-51 leaf sheaths in November across arginine concentrations and pH treatments revealed significant variations. B3 treatment (20 g/mu Arg, pH 7.0) resulted in the highest sucrose content compared to the control (Figure 2A), suggesting that this optimal combination of Arg concentration and pH significantly promotes sucrose accumulation in leaf sheaths. Correspondingly, stem sucrose levels in YZ05-51 treated with 20 g/mu Arg at pH 7.0 reached 18.49% in December–January, compared to 15.58% in the control (Figure 2B), compensating for the defects in sucrose transport ability.

**Figure 3:**
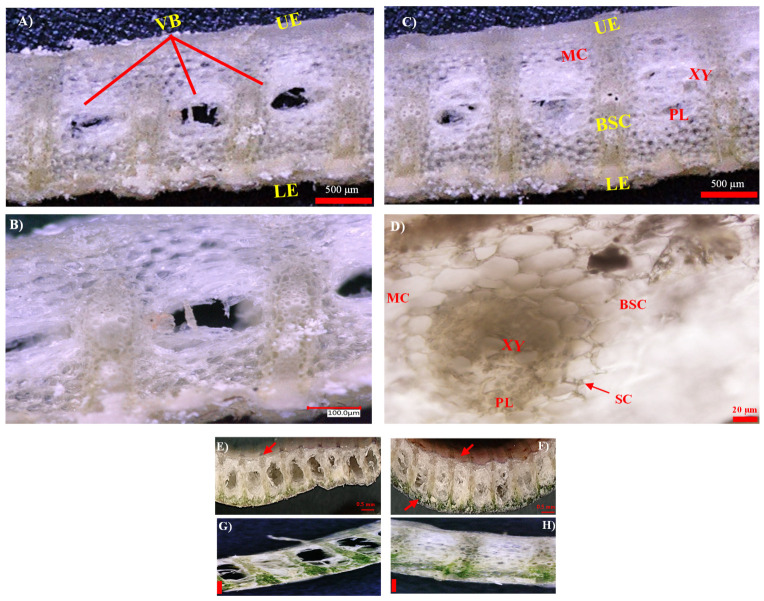
Light microscopy of leaf sheath cross-sections from sugarcane cv. YZ05-51 under control and arginine treatment. CK (**A**,**B**), treated with 20 g/mu Arg, of 7.0 pH (**C**,**D**). Anatomical characteristics and magnification of a cross-section of a sugarcane leaf sheath showing the vascular bundle and associated tissues. UE, upper epidermis; LE, lower epidermis; BSC, bundle-sheath cells; MC, mesophyll cells; PL, phloem; VB, vascular bundles; SC, sclereid cell; and XY, xylem. Structure of the leaf sheath base margin in CK (**E**–**G**) and (**F**,**G**) in treatments with 20 g/mu Arg, at 7.0 pH in the sugarcane cultivar YZ05-51. Scale bar = 500 µm. Note: leaf sheath of YZ05-51 sugarcane (**E**,**F**). Control compared with Arg treated with 20 g/mu at pH 7.0; the arrow indicates different coloration of leaf sheath inner and outer surfaces (**G**,**H**). Structure of the leaf sheath base margin of the sugarcane cultivar YZ05-51 under control conditions and after treatment with 20 g/mu Arg at pH 7.0.

Light microscopy of YZ05-51 leaf sheaths revealed distinct differences between the control and 20 g/mµ Arg treatment groups. Treated leaf sheaths exhibited bright greenish-purple coloration (Figure 3E,F) and significantly greater thickness (Figure 3G,H) compared to the control. Mechanical tissue proportion was higher in treated samples, with a more uniform distribution throughout the leaf sheath, whereas the control showed concentrated mechanical tissue in specific regions (Figure 3A,B). Control samples had larger air cavities with irregular rectangular or circular shapes, whereas treated samples displayed smaller, square-shaped, and more compact air cavities (Figure 3A,B). These structural modifications in leaf sheaths, induced by 20 g/mµ Arg at pH 7.0, are consistent with the early Arg-induced transcriptomic and metabolomic responses observed 6 h post-treatment, and may have contributed to the sucrose accumulation from November to January.

### 2.2. Metabolome Profiling and Differentially Accumulated Metabolites (DAMs)

**Figure 4:**
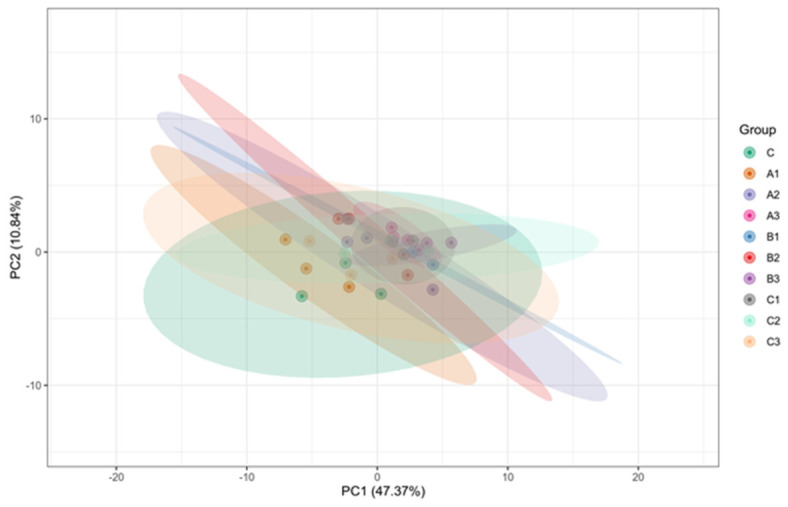
Principal component analysis (PCA) of metabolites in sugarcane cv. YZ05-51 leaf sheaths. Samples include the untreated control (C) and arginine treatments across three pH levels (A: 6.4, B: 7.0, C: 7.4) and three concentrations (1: 100 g/mu, 2: 50 g/mu, 3: 20 g/mu), with three biological replicates per group. The first two principal components (PC1 and PC2) explained 47.37% and 10.84% of the total variance, respectively. Ellipses represent 95% confidence intervals for each treatment group.

To quantify the targeted metabolites in the leaf sheath of YZ05-51 sugarcane under different concentrations of Arg, we adopted targeted metabolomics analysis related to sugar metabolism. A total of 32 metabolites grouped into three main classes were identified from the 30 samples. Among them, 25 monosaccharides, 6 disaccharides, and 1 trisaccharide metabolite were obtained (Supplementary Data S1). The overlay of the TIC plots between different QC samples demonstrated that our data showed high repeatability and reliability (Supplementary Figure S1). Furthermore, principal component analysis (PCA) was performed to visualize metabolite composition across samples. The results revealed that the metabolite composition along PC1 (x-axis) accounted for 47.37%, and PC2 (y-axis) represented 10.84% of the variability. The analysis also showed that the metabolite composition in the leaf sheath of the control samples was clustered, whereas the metabolite composition in the leaf sheath of the YZ05-51 variety exhibited a contrasting pattern across all treatments (Figure 4). Hierarchical cluster analysis (HCA) further highlighted that the trends in metabolite composition varied significantly with different pH levels and Arg concentrations (Supplementary Figure S2). However, the metabolite compositions associated with pentose and glucuronate interconversions and ABC transporters were relatively consistent.

**Figure 5:**
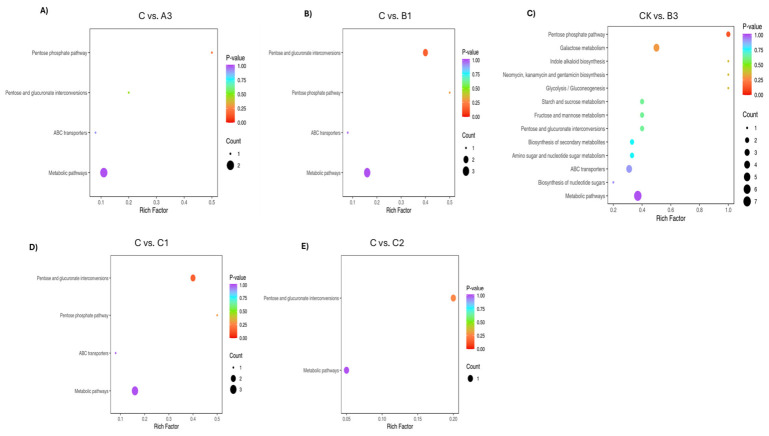
KEGG pathway enrichment analysis of differentially expressed metabolites (DEMs) in YZ05-51 sugarcane leaf sheaths under different arginine treatments. Bubble plots illustrate the functional enrichment of DEMs for five pairwise comparisons: (**A**) C vs. A3; (**B**) C vs. B1; (**C**) CK vs. B3; (**D**) C vs. C1; and (**E**) C vs. C2. Each bubble represents a distinct metabolic pathway, with the x-axis indicating the rich factor (ratio of DEMs annotated to the pathway relative to the total background metabolites), bubble size corresponding to the number of DEMs mapped to the pathway, and color gradient denoting the significance of enrichment (*p*-value, from low to high significance).

To identify significantly differentially expressed dead-end metabolites (DEMs) associated with increased sucrose content, the variable importance in projection (VIP) ≥ 1.0 together with fold change ≥ 2 or ≤0.5 were adjusted as the thresholds. A total of 24 DEMs were identified across nine groups compared with the control group (CK vs. A1, CK vs. A2, CK vs. A3, CK vs. B1, CK vs. B2, CK vs. B3, CK vs. C1, CK vs. C2, and CK vs. C3) (Supplementary Data S2), with most showing significant downregulation of xylulose in CK vs. A3, xylulose in CK vs. B1, xylulose, mannose, glucose, galactose, fructose, arabinitol, and ribose in CK vs. B3. Notably, several metabolites, such as xylulose, mannose, and ribose, were significantly downregulated in CK vs. C1 comparisons. However, arabinitol was downregulated only in the CK vs. C2 group. These results imply that DEMs related to monosaccharide, disaccharide, and trisaccharide biosynthesis are likely to play important roles in different treatments of the sugarcane YZ05-51 variety. We also identified that these DEMs were from distinct classes and constituted the pentose phosphate pathway, pentose and glucuronate interconversions, and ABC transporters. These data suggest that a variety of mono-, di-, and trisaccharides were involved in different concentrations of L-Arg (20, 50, and 100 g/mu) with three different pH levels (6.4, 7.0, and 7.4). KEGG pathway analysis of the significant DEMs revealed notable enrichment in several key pathways, including the pentose phosphate pathway, starch and sucrose metabolism, fructose and mannose metabolism, and ABC transporter pathways, across the compared groups (Figure 5 and Supplementary Data S3). These results imply that DEMs related to metabolic pathways, the pentose phosphate pathway, starch, and sucrose metabolism are likely to play important roles in enhancing sucrose content in YZ05-51 sugarcane.

### 2.3. Transcriptomic Responses of YZ05-51 Sugarcane Under Arg

**Figure 6:**
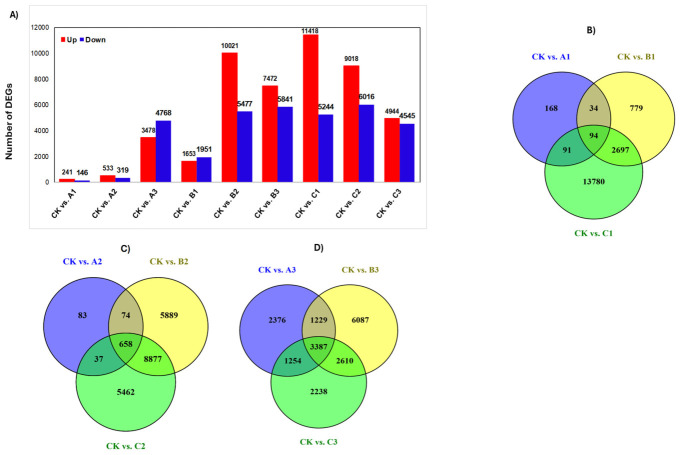
Expression profiles of pH level-responsive genes at different concentrations of Arg. (**A**) Number of differentially expressed genes (DEGs) in each comparison. CK vs. A1, vs. CK vs. B1, and CK vs. C1 represent the differentially expressed genes under 100 g/mu of Arg, and control comparisons. CK vs. A2, vs. CK vs. B2, and CK vs. C2 represent the differentially expressed genes under 50 g/mu, of Arg, CK vs. A3, vs. CK vs. B3, and CK vs. C3 represent the differentially expressed genes under 20 g/mu, of Arg, (**B**–**D**). The Venn diagram displays the relationships between differentially expressed genes (DEGs) of various treatments (**B**) CK vs. A1, vs. CK vs. B1, and CK vs. C1 (**C**) CK vs. A2, vs. CK vs. B2, and CK vs. C2 (**D**) CK vs. A3, vs. CK vs. B3, and CK vs. C3, respectively.

To gain a deeper understanding of the molecular basis underlying the metabolic differences observed at various Arg concentrations and pH levels, we conducted whole-genome transcriptome sequencing using leaf sheath tissues. A total of 233.2 Gb of clean reads were generated from the 30 libraries after removing adaptor sequences and low-quality reads. The percentage of high-quality reads with a Q30 score > 97.38%, GC content varied from 53.95% to 57.30%, and the successfully mapped reads were >88.09% (Supplementary Data S4). The coefficient of correlation between the biological replicates of the same tissues was >0.86 (Supplementary Figure S3). These results indicate the high quality of the sequencing data. Furthermore, DEGs were identified via pair-wise comparison with the control group (CK vs. A1, CK vs. A2, CK vs. A3, CK vs. B1, CK vs. B2, CK vs. B3, CK vs. C1, CK vs. C2, and CK vs. C3). The analysis revealed that CK vs. C1 had the largest number of DEGs (16,662), of which 11,418 were upregulated and 5244 were downregulated (Figure 6). A lower number of DEGs was identified in CK vs. A1 (387), of which 241 were upregulated and 146 were downregulated. Similarly, the number of DEGs under CK vs. A2 revealed 852 DEGs, of which 533 were upregulated and 319 were downregulated. The comparison of CK vs. B2 resulted in 15,498 DEGs, including 10,021 upregulated and 5477 downregulated genes. The comparison of CK vs. C2 revealed a total of 15,034 DEGs, among which 9018 were upregulated and 6016 were downregulated. More detailed information about the diversity of DEGs is provided in Supplementary Data S5.

The Venn diagram illustrates the interaction of DEGs across various Arg concentrations and pH levels (Figure 6B and Supplementary Data S5). Only 94 common DEGs were detected under CK vs. A1, CK vs. B1, and CK vs. C1 (Figure 6B). Notably, 658 common DEGs were recognized among the three comparisons (CK vs. A2, CK vs. B2, and CK vs. C2). Moreover, 3387 genes were found in the comparison of CK vs. A3, CK vs. B3, and CK vs. C3 (Figure 6C,D). These genes are potential candidates involved in common mechanisms regulating sucrose and secondary metabolite production in response to exogenous Arg, which significantly increased sucrose content in the YZ05-51 sugarcane leaf sheath and subsequently in the stem. The DEG analysis across different comparison groups revealed notable variations in gene expression profiles depending on the Arg concentrations and pH levels applied to the YZ05-51 sugarcane variety.

### 2.4. Integrated Metabolomics and Transcriptomic Analysis

**Figure 7:**
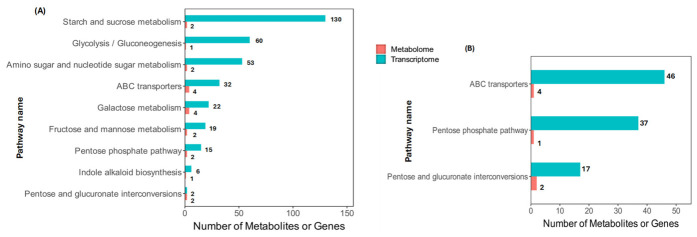
Integrated analyses of the identified differentially expressed genes (DEGs), differentially accumulated metabolites (DAM), and KEGG-enriched pathways. (**A**) CK vs. B3 and (**B**) CK vs. C1.

An integrated analysis of KEGG enrichment pathways, functional analysis, and correlations between the metabolomic and transcriptomic datasets was conducted to minimize false-positive results typically associated with single-omics data. All DEMs and DEGs were mapped to the KEGG pathway database to identify key biological pathways to better understand the relationship between important genes and metabolites. KEGG enrichment analysis showed that 2, 2, 9, 3 and 1 KEGG pathways (*p* < 0.05) were enriched in at least one omics data type in the CK vs. A3, CK vs. B1, CK vs. B3, CK vs. C1, and CK vs. C2 pairwise comparison groups, respectively (Figure 7 and Supplementary Data S6). The DEGs and DEMs were mainly enriched in starch and sucrose metabolism, glycolysis/gluconeogenesis, amino sugar and nucleotide sugar metabolism, ABC transporters, galactose metabolism, fructose and mannose metabolism, pentose phosphate pathway, indole alkaloid biosynthesis, and pentose glucuronate interconversions. Furthermore, five highly enriched pathways, including starch and sucrose metabolism (ko00500), glycolysis/gluconeogenesis (ko00010), amino sugar and nucleotide sugar metabolism (ko00520), ABC transporters (ko02010), and galactose metabolism (ko00052), were selected for subsequent analysis to explore the potential links between the metabolome and transcriptome datasets. Ultimately, the expression of 16 genes selected from the five highly enriched pathways was validated using qRT-PCR. The results demonstrated a high degree of consistency between the transcriptome and qRT-PCR results (Supplementary Figure S4A–D).

### 2.5. DEGs and DAMs Involved in Starch and Sucrose Metabolism

To better understand the relationship between the identified genes and metabolites, the DEGs and DAMs from nine different comparisons were successfully mapped to KEGG pathways. In the KEGG mapping analysis of CK vs. B3, nine pathways were found to be highly enriched compared with the CK vs. C1 group (Figure 7A,B). In the CK vs. B3 group, a total of 130 genes and two key metabolites were simultaneously mapped to the starch and sucrose metabolism pathway, and 60 genes and one key metabolite from the glycolysis/gluconeogenesis (ko00010) pathway. Two key metabolites and 53 genes were simultaneously mapped to amino sugar and nucleotide sugar metabolism (ko00520), and four key metabolites and 32 genes for ABC transporters (ko02010) (Figure 7A and Supplementary Figure S5A–D). These results further emphasize the importance of exploring the gene regulatory dynamics of sucrose production in YZ05-51 sugarcane under optimal conditions of 20 g/mu Arg and pH 7.0.

KEGG enrichment analysis revealed that several DEGs were significantly enriched in starch and sucrose metabolism (map00500) pathways (Figure 7A, Supplementary Figure S5A and Data S7). The starch and sucrose metabolism genes differed among CK vs. A3, CK vs. B1, CK vs. B3, CK vs. C1, and CK vs. C2 comparisons. Several key genes were significantly upregulated, including 1,4 α-glucan branching enzyme/starch branching enzyme (SBE) (ID: SoffiXsponR570.7_10Ag171000. v2.1), biofilm formation–like protein (ID: SoffiXsponR570.06Fg122000.v2.1), glycogen phosphorylase (PYGL) (ID: SoffiXsponR570.01Ag086500.v2.1), GDP-mannose pyrophosphorylase/mannose-1-phasphte guanylyltransferase (GMPPA), trehalose-6-phosphate synthase (TPS1) (ID: SoffiXsponR570.03Dg233500.v2.1), α-amylases (ID: SoffiXsponR570.07Cg062400.v2.1), cyanoamino acid metabolism/degradation of flavonoids/biosynthesis for plant secondary metabolites (ID: SoffiXsponR570.02Ag296500.v2.1), β-glucosidase lactase phlorizin hydrolase (LPH) (ID: SoffiXsponR570.7_10Ag243800. v2.1), and ribokinases (RBKS) (ID: SoffiXsponR570.04Cg101600.v2.1) under CK vs. B3, of 20 g/mu Arg at pH 7.0, respectively. The high expression of these genes indicates that the leaf sheaths of sugarcane exhibit different physiological response mechanisms under different pH values and concentrations. Moreover, the response of the CK vs. B3 group in terms of sucrose synthesis and accumulation was superior to that of the other groups. Collectively, enzymes such as α-amylase and branching enzymes facilitate the breakdown of starch, whereas glycogen phosphorylase and β-glucosidase help mobilize glucose, ultimately converting stored carbohydrates into sucrose. Additionally, TPS1 enzymes play a crucial role in maintaining the metabolic balance between starch and sucrose, responding to both internal signals and external environmental factors. Moreover, ID: SoffiXsponR570.7_10Ag383700.v2.1, ID: SoffiXsponR570.07Eg227000.v2.1, ID: SoffiXsponR570.03Cg038700.v2.1, and ID: SoffiXsponR570.01Dg308300.v2.1, related to D-glucose and D-fructose metabolites, exhibited downregulated expression in CK vs. B3 (Figure 2A,B, Supplementary Figure S5A, and Supplementary Data S7). The downregulation of D-glucose and D-fructose metabolism-related genes in CK vs. B3 suggests a metabolic shift towards sucrose accumulation rather than sugar utilization for other metabolic activities, especially under 20 g/mu Arg at pH 7.0.

Integrated transcriptomic and metabolomic KEGG analysis further identified several DEGs that were significantly enriched in key pathways, including glycolysis/gluconeogenesis and the tricarboxylic acid (TCA) cycle. Notably, under the CK vs. B3 condition, many genes associated with starch/carbohydrate metabolism were downregulated. To gain deeper insights into sucrose metabolism, particularly in the leaf sheath of YZ05-51, which accumulated high sucrose content, we further investigated genes associated with glycolysis/gluconeogenesis (ko00010) (Supplementary Figure S5B and Data S7). These genes are likely to play a crucial role in regulating sucrose content during the later developmental stages in December and January (Figure 2A,B). The metabolic pathways exhibited a strong response to Arg treatment (20 g/mu, pH 7.0), which aligned with the transcriptomic data (Supplementary Data S5). This treatment highlighted differential gene expressions in the CK vs. B3 condition (Figure 7A, Supplementary Figure S5B). For example, two key genes, ID: SoffiXsponR570.5_9Ag134600. v2.1, which is involved in the TCA cycle, and ID: SoffiXsponR570.02Dg312500.v2.1, encoding phosphoglycerate mutase (PGAM2), were upregulated, suggesting a potential role in enhancing metabolic flux toward energy production and sugar accumulation. Conversely, ID: SoffiXsponR570.05Bg230600.v2.1; triosephosphate isomerase (TPI) was downregulated in the CK vs. B3. Nevertheless, these genes were not detected in other comparisons of YZ05-51 treatment with different Arg concentrations and pH levels.

Additionally, multiple genes, including ID: SoffiXsponR570.02Gg045600.v2.1 (dihydrolipoamide dehydrogenase (DLD), ID: SoffiXsponR570.04Gg106700.v2.1 (3-phosphoglycerate kinase (PGK1), and ID: SoffiXsponR570.6_9Ag059600. v2.1, fructose-biphosphate aldolase (ALDOA) was downregulated under various treatment conditions. This downregulation contributes to the slowing of glycolysis/gluconeogenesis and the tricarboxylic acid (TCA) cycle, ultimately reducing the energy supply and limiting the production of essential metabolic intermediates for sucrose accumulation. More importantly, the downregulation of DLD and PGK1 suggests that after the leaf sheaths were initially exposed to different pH values or a high concentration of Arg, the cells of the leaf sheaths generated an emergency physiological response and adjusted the metabolic direction by downregulating the expression of these genes to adapt to environmental changes and allocate more energy and substances to other physiological processes, such as coping with stress or maintaining ionic balance within the cells.

### 2.6. KEGG Enrichment of Amino Sugar and Nucleotide Sugar Pathway

KEGG enrichment analysis demonstrated that several DEGs and DAMs were significantly enriched in amino sugar and nucleotide sugar metabolism pathways (Figure 7A, Supplementary Figure S5C). Notably, this pathway was observed in the CK vs. B3, with no significant changes detected in other treatment comparisons. Specifically, the genes ID: SoffiXsponR570.09Eg027800 and ID: SoffiXsponR570.01Fg183100 were upregulated, indicating enhanced capacity for sugar metabolism, which may be crucial for cell wall formation or glycosylation, both of which play an essential role in plant growth and development, and may enhance the transport of sucrose or may be indispensable carriers for sucrose synthesis and transport. Additionally, the upregulation of ID: SoffiXsponR570.10Dg130200, which encodes mannose-6-phosphate isomerase (MPI), suggests improved energy flow through glycolysis, thereby contributing to the sugarcane plants’ overall metabolic efficiency. Similarly, ID: SoffiXsponR570.06Cg041100, which encodes the TIM10/TIM12 mitochondrial import complex, was upregulated, indicating increased mitochondrial activity and energy production, which are vital for supporting overall metabolic functions. Furthermore, the upregulation of ID: SoffiXsponR570.07Bg124500, a chitinase-like protein, suggests that Arg treatment promotes cell wall fortification by supporting polyamine synthesis, which may contribute to the development of robust leaf sheaths (Figure 3D).

The integrated KEGG analysis also provided evidence of enhanced amino sugar and nucleotide sugar metabolism through the downregulation of ID: SoffiXsponR570.05Ag038400.v2.1, which encodes UDP-glucose 4-epimerase/UDP-sulfoquinovose synthase, in the CK vs. B3 group, with no significant changes observed in the other groups. Additionally, several genes encoding putative enzymes related to amino and nucleotide sugar metabolism were downregulated in the CK vs. B3 comparison, whereas their expression remained unchanged in the other treatment groups (Supplementary Data S7). Similarly, 21 genes were downregulated in galactose metabolism, and these genes were found to be responsible for four metabolites, except for one gene: pyrophosphate-dependent phosphofructo-1-kinase (PPi-PFKs; ID: SoffiXsponR570.1Z272500.v2.1), which was upregulated (Supplementary Data S7). This phenomenon may be correlated with starch and sucrose metabolism and glycolysis/gluconeogenesis or carbon metabolism.

### 2.7. KEGG Enrichment of ABC Transporters

Sucrose-related metabolite and transcriptomic analyses were conducted to further investigate the ABC transporter mechanism in the YZ05-51 sugarcane leaf sheath within four DEMs, including mannose, glucose, fructose, and ribose, which significantly decreased under CK vs. B3, while sucrose content improved (Figure 2A,B and Figure 3C,D). ATP-binding cassette (ABC) transporters are a ubiquitous superfamily of integral membrane proteins that transport substances across biological membranes, including peptides. The changes in the expression levels of these genes were consistent with the changes in the levels of related metabolites regulated by the enzymes encoded by the genes, which can be used to verify the reliability of the transcriptional data. For instance, SoffiXsponR570.07Dg180700.v2.1; peptide exporter, ID: SoffiXsponR570.7_10Ag319200.v2.1 (MXR) is a member of the ABC transporter and ID: SoffiXsponR570.7_10Ag319200.v2.1; multidrug/pheromone exporter upregulated under CK vs. B3, of 20 g/mu Arg at pH 7.0 (Figure 7A, Supplementary Figure S5D and Data S7). These transporters may play a crucial role in moving a wide range of substrates across cellular membranes using ATP to transport various biological molecules, including peptides. This phenomenon may be linked to the activation of sucrose transport in the phloem of leaf sheath tissue and the enhancement of sucrose content from October to January compared with the control (Figure 2 and Figure 3).

## 3. Discussion

After spraying three sugarcane varieties with different sucrose metabolic types with L-arginine hydrochloride, the results showed that the three varieties with different metabolic types had certain differences in sucrose accumulation. Among them, low-concentration L-arginine hydrochloride had a sugar-increasing effect on YZ05-51, which is prone to defoliation. This genotype-dependent response highlights the importance of matching chemical regulators to variety characteristics, which is an efficient strategy for sucrose improvement, especially given the slow progress of conventional breeding. Aiming at the differences in sucrose synthesis, transport, and decomposition activities during sucrose metabolism among varieties, applying corresponding reagents can effectively increase sugarcane sucrose content. The YZ05-51 sugarcane cultivars exhibit distinctive agronomic characteristics that distinguish them from recently developed fifth-generation sugarcane varieties [19,20]. Its advantages include rapid seedling emergence, enhanced stress resistance, and adaptability to harsh environmental conditions, making it an ideal choice for cultivation on dry slopes [19,21,22]. In contrast, YZ08-1609 may possess less responsive arginine pathways or reduced transporter activity, thereby limiting the influence of arginine on sucrose metabolism. The compromised condition of YT93-159, characterized by mosaic virus infection and diminished photosynthesis, likely impairs its arginine metabolism or response, resulting in an absence of sucrose enhancement. However, sucrose accumulation in sugarcane is an intricate process controlled by environmental and endogenous factors. The improvement of sucrose content through conventional breeding has plateaued or progressed at a comparatively slow rate [23]. Consequently, achieving significant advancements in sugarcane crossbreeding remains a challenging task, posing a significant obstacle for sugarcane breeding programs [7,23,24,25,26]. Inducing the carbon source metabolic flux specifically in YZ05-51sugarcane with exogenous reagents to promote sucrose yield is an effective strategy. In the present investigation, the control (CK) recorded a sucrose yield of 15.58% in January, while the 20 g/mu arginine treatment reached 18.49%, representing an increase of 2.91 percentage points (a relative improvement of approximately 18.68%) compared with the control (Figure 2A,B). This further indicates that under the induction of low-dose arginine hydrochloride, some sugarcane germplasms can increase sucrose content [18].

Arg induces the synthesis of nitric oxide (NO), proline, and polyamines (PAs) in plants. NO can be produced via various pathways, including Arg-dependent mechanisms [14]. However, nitric oxide synthase (NOS) genes have not yet been identified in higher plants. Furthermore, arginine-succinate lyase complicates citrulline synthesis from Arg, thereby affecting NOS activity. A reduction in leaf S-nitrosothiol (RSNO) levels under water deficit conditions was less pronounced in Arg-supplied sugarcane plants [27,28]. This observation suggests that NO may enhance carboxylation during photosynthesis in sugarcane under drought conditions, with Rubisco as a potential target for S-nitrosation, thus improving photosynthetic rates in Arg-treated plants [15,29]. Furthermore, Arg-treated barley exhibited increased morphological growth characteristics under drought stress conditions [30]. The application of (1 mM) arginine significantly improved the biomass of sunflower plants [31]. Moreover, pretreatment with Arg alleviated the effects of drought on wheat plant growth and development [32]. These results might be due to the conversion of L-arginine into proline and nitric oxide, which is essential for plant responses to drought stress. The role of arginine in counteracting the adverse effects on plants may be due to the production of polyamines, which contribute to a wide range of biological processes, such as growth, biochemical metabolism, and abiotic stress [33]. Similarly, in our study, L-Arg at different concentrations and pH levels influenced sucrose metabolism in the YZ05-51 sugarcane variety. Furthermore, our integrated metabolomic and transcriptomic analyses provided a deeper understanding of YZ05-51 sugarcane plants, which adjust metabolic processes in response to varying external conditions in the presence of Arg at different concentrations and pH levels.

Elucidating the regulatory mechanism(s) and identifying the key genes and targeted metabolites controlling sucrose accumulation in sugarcane are essential for sucrose improvement through transgenics, genome editing, or marker-assisted selection (MAS) breeding [34,35,36,37]. However, the high-sugar phenotypes observed in these studies have not been consistently replicated under field conditions, indicating the genetic and regulatory complexity underlying this trait. In the present study, metabolomic and transcriptomic changes associated with sucrose accumulation during maturity, specifically in YZ05-51 sugarcane with significant differences in sucrose content (Figure 2A,B) were examined. Sucrose accumulation at different pH levels was experimentally manipulated by externally applying L-Arg to better resolve the molecular changes associated with sucrose accumulation.

Metabolomics is an effective approach for measuring the metabolite compositions of various plant tissues. Targeted and untargeted metabolomics techniques have also been used to identify metabolites present in different organs of plant species during various developmental stages [38]. In the present study, we used targeted metabolomics to identify 32 metabolites, including monosaccharides, disaccharides, and trisaccharides, involved in the sucrose metabolic pathway. Notably, the concentrations of L-Arg and pH influenced the accumulation of sugar-related metabolites. For instance, monosaccharides such as glucose, mannose, and fructose were significantly downregulated in specific groups treated with higher Arg concentrations, particularly at basic or acidic pH values, compared with the control pH level. The metabolic flux of carbon sources in sugarcane changes after arginine application.

KEGG pathway enrichment analysis further showed that many metabolites displayed downregulation patterns for xylulose mannose, glucose, galactose, fructose, and arabinitol in YZ05-51 sugarcane. Further analysis of the significant DEMs revealed that KEGG pathways, including metabolic pathways, the pentose phosphate pathway, starch and sucrose metabolism, fructose and mannose metabolism, and ABC transporters, were significantly enriched (Figure 5 and Supplementary Data S3). This indicates an enhancement in sucrose transport activity, whereby sugar metabolism-related metabolites in the leaf sheaths are further translocated to the culms for sucrose biosynthesis. Notably, sucrose production was prominent at this stage, contributing to the steady accumulation of metabolites [4,24,39,40]. While sucrose transport activity increases, other sugar content is relatively reduced, stimulating sugar metabolism to sucrose synthesis and transport. Moreover, PCA showed a clear separation of samples based on the treatment conditions, reinforcing the idea that both Arg concentration and pH levels have a substantial impact on sugar metabolism (Figure 4). From the data of co-expressed genes and sucrose content in the Venn diagram, it can be seen that under the condition of low Arg concentration, it is conducive to the flow of sucrose transport and synthesis, and there are more co-expressed genes; while after increasing the Arg concentration, the number of co-expressed genes gradually decreased (Figure 6B–D), this may be due to the fact that after the increase in the concentration of arginine, it had a certain inhibitory effect on the transport of sucrose specifically in YZ05-51 sugarcane [18]. Under acidic and alkaline pH conditions, the cells were in different stages of the defense response; therefore, the number of co-expressed genes decreased. Due to the physiological disorder caused by the high concentration of Arg in the leaf sheath, different pH concentrations trigger physiological responses, which in turn affect sucrose transport and reduce the number of co-expressed genes. Furthermore, HCA also revealed distinct metabolite patterns across the treatments, which indicates that nutrient supplementation can lead to cumulative changes in sugar metabolism (Supplementary Figure S2). At the same time, these results show that a decrease in the content of xylulose will lead to a relative increase in the precursor substances involved in sucrose synthesis, such as triose phosphates. These precursor substances can ultimately form sucrose through a series of enzymatic reactions. Furthermore, the decrease in xylulose can affect the relevant signaling pathways, activate key enzymes involved in sucrose transport, and promote sucrose transport ability. These results indicate that the leaf sheath is not the main tissue for sucrose synthesis; its energy metabolism exhibits vigorous activity after arginine treatment. This suggests that the leaf sheath, as a transport carrier between the source and sink, is also in a vigorous stage in terms of sucrose transport capacity.

Transcriptome sequencing identified significant DEGs in response to varying Arg concentrations and pH levels (Figure 6). A total of 233.2 Gb of clean reads were generated from 30 libraries (Supplementary Data S4). Interestingly, the number of DEGs was highest in the CK vs. C1 and CK vs. B2 comparison groups, indicating that specific combinations of Arg concentration and pH levels induce more profound transcriptional changes than other combinations. In contrast, the CK vs. A1 group exhibited the lowest number of DEGs (Figure 6). DEGseq was identified as the optimal method for DEG screening. Its reliability was further confirmed by the high concordance with qRT-PCR validation of sixteen genes. Previous studies investigated the sugarcane metabolic and transcript landscapes of seven different varieties and uncovered essential transcriptomic and transcriptional regulatory networks for metabolic changes [41]. A high correlation was also found in pathways related to carbon fixation, starch, and sucrose metabolism [24]. Carbon metabolism, which encompasses the carbon fixation pathway and starch and sucrose metabolism, plays a crucial role in plant growth and biomass accumulation. It also serves as a precursor for synthesizing other essential biomolecules [42,43]. The expression patterns of DEGs associated with the TCA cycle, starch and sucrose metabolism, glycolysis/gluconeogenesis, and other metabolic pathways in this study aligned with expression trends previously reported in high-sugar varieties compared to low-sugar varieties based on available transcriptome data [4,24,25,40,41]. These results suggest that alterations in the expression of these key metabolic genes may be associated with the differential sugar accumulation observed between varieties. Our findings support previous research on sucrose accumulation in sugarcane, emphasizing the potential genetic basis for selecting varieties with enhanced sugar yields. Further investigation of the DEGs related to these pathways could provide valuable insights into breeding programs aimed at improving sugar content (Figure 7).

Existing evidence underscores the complexity of sugar metabolism and accumulation mechanisms in sugarcane. Our integrated metabolomic and transcriptomic analyses provide a comprehensive understanding of the influence of Arg on sugar metabolism. Key metabolic pathways, including starch and sucrose metabolism, glycolysis/gluconeogenesis, amino sugar and nucleotide sugar metabolism, and ABC transporters, were predominantly enriched in the CK vs. B3 comparison under the 20 g/mu Arg treatment at pH 7.0 (Figure 5 and Figure 7). Compared to the other treatment groups, this enrichment suggests that these metabolic pathways play a crucial role in sucrose transport and biosynthesis in YZ05-51. The upregulation of key genes involved in energy production and sugar metabolism further emphasizes metabolic efficiency under these specific conditions. The enrichment of ABC transporters can enhance the sucrose transport activity of the cell membrane, specifically in YZ05-51 sugarcane leaf sheaths and improve the transport efficiency of sucrose from the source to the sink. Xylulose, mannose, glucose, galactose, fructose, arabinitol, and ribose were downregulated, which indirectly indicates that during the process of improving sucrose transport efficiency, the sucrose synthesis capacity of the leaf sheath is enhanced simultaneously, whereas the content of other free monosaccharides and oligosaccharides is also increased. The coordination between gene expression and metabolic flux indicates that Arg plays a crucial role in enhancing sugarcane’s capacity to store sucrose, especially under optimal conditions. For instance, genes related to sucrose metabolism, such as *SBE*, *α-amylases*, *PYGL*, *GMPPA*, *MGAM*, *TPS1*, *LPH*, and *RBKS*, were upregulated, whereas *MGAM* family border membrane enzymes and several other genes were downregulated (Supplementary Data S7), indicating that arginine treatment enhanced metabolic efficiency, supporting the increased demand for sugar synthesis and storage. Simultaneously, expression of these genes also resulted in modifications in the levels of specific metabolites, such as glucose and fructose (Figure 7A). *SBEs* play a critical role in determining the structure and properties of starch and can promote the formation of branches in amylose, thereby enabling more energy storage. Additionally, α-amylases can facilitate the hydrolysis of starch into small-molecule carbohydrates. These findings indicate that after L-arginine hydrochloride treatment, the genes related to starch synthesis and catabolism in sugarcane leaf sheaths are actively expressed, and the tissue exhibits vigorous activity. This is conducive to promoting sucrose transport in leaf sheaths, which act as intermediates between source and sink organs. The trehalose-6-phosphate synthase gene (*TPS1*) can catalyze the synthesis of sucrose-signaling metabolites and influence carbohydrate metabolism; increased expression of this gene also helps promote sucrose synthesis, providing more precursor substances for sucrose accumulation in sugarcane stems [44,45].

To further support this notion, numerous genes associated with glycolysis, including fructose-bisphosphate aldolase (*FBA* or aldolase *ALD*), were downregulated. FBA plays a vital role in signal transduction and carbohydrate metabolism in different plant species. Aldolase activity is also involved in glycolytic and gluconeogenic reactions. These reactions are involved in carbon fixation and sucrose metabolism in the chloroplast stroma and the cytosol of green plants [39,46,47]. This observation suggests that under certain conditions, YZ05-51 sugarcane shows a specific positive correlation between early (November) and late (December) stages, prioritizing sucrose storage (Figure 2A,B) over immediate energy production. This strategy may serve as an adaptive mechanism for optimizing carbohydrate reserves. Interestingly, these results suggest that the genes and metabolites involved in carbon metabolism pathways exhibit distinct expression patterns. However, additional research is required to confirm the pathways that promote high-sucrose performance in YZ05-51 sugarcane.

Integrated KEGG pathway analysis further explored amino sugar and nucleotide sugar metabolism, which was more responsive in the CK vs. B3 group compared to the other treatments. Key genes and metabolites, including glucose and mannose, were mapped to the metabolic pathways (Figure 7, Supplementary Data S7). For instance, genes encoding *MPI* can be physically linked to glucose and mannose metabolism [48]. The *TIM10/TIM12* complex functions as a mitochondrial carrier protein system, facilitating the sorting of substrates into mitochondrial sub-compartments, mediated by translocase enzymes located in the outer and inner mitochondrial membranes [49,50,51]. The upregulation of these genes in the YZ05-51 leaf sheath increased mitochondrial activity and energy production, thereby supporting overall metabolic functions. UDP-glucose 4-epimerase/UDP-sulfoquinovose synthase (UGPase) catalyzes the production of UDP-glucose, a key precursor for sucrose and polysaccharide metabolism, as well as cell wall biosynthesis [52,53]. This finding suggests a strengthened cellular structure (Figure 3D). Notably, the upregulation of these genes enhanced the capacity of YZ05-51 for sucrose accumulation under optimal conditions.

ABC transporters represent a diverse superfamily of integral membrane proteins that facilitate the transport of various molecules, including peptides, across biological membranes [54,55,56]. Alterations in the expression levels of ABC transporter genes are associated with related metabolites, such as mannose, glucose, fructose, and ribose, and are regulated by these ABC transporters, such as the peptide exporter and MXR exporter under optimal Arg conditions (Figure 7A, Supplementary Figure S5D; Supplementary Data S7). These transporters are crucial for the translocation of a wide range of substrates across cellular membranes utilizing ATP, thereby facilitating the transport of various biological molecules, including peptides, and improving sucrose transport and accumulation in YZ05-51 sugarcane. These findings are specific to the YZ05-51 sugarcane genotypes. Further, mechanistic interpretations are correlative and hypothesis-driven, relying exclusively on multi-omics data. To substantiate these proposed mechanisms, future research should incorporate direct functional validation.

## 4. Materials and Methods

### 4.1. Plant Material and Experimental Conditions

Three commercial sugarcane varieties—YZ05-51, YT93-159, and YZ08-1609—were cultivated in Gengma, Lancang, and Menglian counties, Yunnan Province, China, a major sugarcane-producing region. The study adopted a split-plot experimental design with three independent locations, serving as site replication. Each independent 1-mu field plot was an experimental unit for treatment, phenotypic measurement and tissue sampling; each treatment had three 1-mu plots/sites as biological replicates. In February 2022, each variety was planted in a 20-mu area (1 mu = 0.0667 ha) at each experimental site, adhering to standardized agronomic practices for commercial sugarcane production. In November 2022, as sugarcane was at a late growth stage, foliar L-arginine spraying treatments were conducted on the three sugarcane varieties across all experimental sites. All spraying solutions were adjusted to a uniform pH of 7.0, and three application doses of 20, 50 and 100 g/mu were set, with each treatment covering 3 mu, with each treatment distributed into three independent 1-mu field plots per site (biological replicate). Each treatment solution was prepared by dissolving the respective mass of L-arginine hydrochloride (purity ≥ 99%, Sigma-Aldrich, St. Louis, MO, USA) in 10 L of deionized water, following a modified method [15]. Unsprayed plots were set as the control group (CK). To avoid cross-contamination between treatments, adjacent plots (including treatment and control plots) were separated by about a 1-mu buffer zone with no sugarcane planting. Foliar spraying was conducted using an agricultural drone (DJI Agras T60, DJI Technology Co., Ltd., Shenzhen, China), flying at a height of 3 m above the sugarcane canopy (the average plant height of sugarcane at the treatment stage was approximately 2.7 m) and a constant flight speed of 3 m/s, ensuring uniform coverage of the treatment solution (30 L per 3-mu plot). The control group was sprayed with an equal volume of deionized water under the same spraying conditions to eliminate the confounding effect of water spraying. In statistical analysis, three planting sites were site-level replicates, and three 1-mu subplots per treatment at each site were plot-level biological replicates for integrated analysis. Hierarchical variance analysis partitioned variance among sites, plots within sites, and treatments; multiple comparison tests assessed differences between control and arginine groups.

### 4.2. Arginine Treatment and Multi-Omics Sampling

In November 2023 (as consistent with the late growth stage Arg treatments), additional foliar Arg treatments and tissue sampling were conducted. Based on preliminary repeated experiments, YZ05-51 was selected as the L-arginine-responsive sugarcane genotype for multi-omics analysis. A standardized spraying and sampling protocol was established to ensure the reliable detection of sugar metabolism-related gene expression: foliar spraying of L-arginine hydrochloride commenced at 9:00 a.m. after complete dew dissipation, and tissue sampling was completed before 3:00 p.m. This timing avoided the confounding effect of weakened photosynthetic activity in the late afternoon and ensured adequate and stable expression of genes associated with sugar metabolism following Arg application. The experiment included a control group (CK, sprayed with deionized water) and three gradient Arg concentrations (20, 50, and 100 g/mu) across three pH levels (6.4, 7.0, and 7.4), resulting in a total of 10 treatment groups (3 pH levels × 3 Arg concentrations + 1 CK), with each treatment covering 1 mu. The pH of each treatment solution was adjusted using Tris-NaOH and citric acid buffers and calibrated with a digital pH meter (accuracy ±0.01, Mettler Toledo, Zurich, Switzerland). Arg spraying was meticulously performed to prevent cross-contamination between treatments. The four core treatment groups were designated as; C (control), A (pH 6.4), B (pH 7.0), and C (pH 7.4); each group was further subdivided by Arg concentration (100, 50, and 20 g/mu), labeled as A1/A2/A3, B1/B2/B3, and C1/C2/C3, respectively. The upper +3 mature leaf sheaths were collected from YZ05-51 plants at 6 h post-treatment. Each treatment group included three biological replicates. All collected samples were immediately frozen in liquid nitrogen to preserve RNA and metabolite integrity, then stored at −80 °C until RNA extraction and metabolomic analysis.

### 4.3. Light Microscopy and Sucrose Content Analysis

The upper mature leaf sheath tissues from sugarcane plants subjected to different treatments were selected for microscopic observations. Notably, as YZ05-51 samples with the most significant phenotypic differences were prioritized for microscopic examination, while no obvious differences were detected in YZ08-1609 and YT93-159. The ultrastructure of the leaf sheath tissues from both control and Arg-treated plants with different concentrations and pH levels was examined using (Keyence VHX-6000, Keyence Corporation, Osaka, Japan) microscope. Image-Pro Plus 6.0 analysis software was used to observe the air cavity area; the leaf sheaths were cut into 8–10 μm sections using a (HHQ-3658, Huahai, Sci. & Edu., Jinhua, Zhejiang, China) rotatory microtome, and the corresponding tissue area was observed. To assess agronomic and quality-related phenotypic traits, five mature stalks, free from diseases and pests, were randomly selected as a sample replicate selected from each independent field plot across all experimental sites. Sucrose content, Brix degree, fiber content, and reducing sugar content were determined using our established protocol [11]. To dynamically quantify sucrose accumulation, the optical rotation method was employed to measure sucrose concentrations in stalks harvested in December 2022 and January 2023, adhering to the same procedure [11].

### 4.4. Targeted Metabolite Extraction, Quantification and Profiling

The frozen leaf sheath samples were retrieved from storage and subsequently ground into powder using a grinding machine at 30 Hz for 1.5 min. The powdered samples were then analyzed using a GC-MS (Agilent Technologies Co., Ltd, 8890-5977B, Beijing, China). Approximately 20 mg of the powder was weighed and dissolved in 1.2 mL of extraction solution (methanol: isopropyl alcohol: water is 3:3:2), vortexed for 3 min, and then placed in an ultrasonic water bath at 4 °C for 30 min. Next, the samples were centrifuged at 4 °C and 12,000 rpm for 3 min, absorb 50 μL supernatant, add 20 μL internal standard solution with a concentration of 1000 μg/mL, then perform nitrogen blowing and freeze-drying; add 100 μL of methoxylamine hydrochloride pyridine (with a concentration of 15 mg/mL) and incubate at 37 °C for 2 h. Next, 100 μL of N, O-bis (trimethylsilyl) trifluoroacetamide (BSTFA) was added to the samples and incubated at 37 °C for 30 min to obtain the derivatized solution. A 50 μL aliquot of the derivatized solution was transferred, diluted with n-hexane to a final volume of 1 mL, and filtered through a 0.22-μm filter membrane. The filtrate was stored in a brown sample injection vial for GC–MS analysis [40]. The specific chromatographic and mass spectrometric conditions are highlighted in Supplementary Data S8. Pooled quality control (QC) samples were prepared by mixing equal volumes of all experimental samples and analyzed intermittently during the GC–MS run. The coefficient of variation (CV) of metabolite peak intensities across QC samples was calculated, and only metabolites with CV < 30% were retained for further analysis to ensure analytical stability and reproducibility. Standard solutions with different concentrations (0.001 μg/mL, 0.002 μg/mL, 0.005 μg/mL, 0.01 μg/mL, 0.02 μg/mL, 0.05 μg/mL, 0.1 μg/mL, 0.2 μg/mL, 0.5 μg/mL, 1 μg/mL, 2 μg/mL, 5 μg/mL, 10 μg/mL, 20 μg/mL, 50 μg/mL) were prepared. These solutions were used to generate the mass spectrometry peak intensity data corresponding to the quantitative signals for each concentration of the standard products.

Standard curves for different substances were constructed by plotting the concentration ratio of the external standard to the internal standard on the x-axis and the peak area ratio of the external standard to the internal standard on the y-axis. The integral peak area ratios of all detected samples were then substituted into the corresponding linear equations of the standard curves. These values were subsequently used in the appropriate calculation formula to determine the content of the substance in the actual samples. The calculation formula is as follows: content of saccharides (mg/g) = c × V1 × V2/V3/m/1,000,000, where “c” represents the concentration value (μg/mL) obtained by substituting the integral peak area ratio of the sample into the standard curve, “V1” represents the volume of the solution used for constant volume (μL), “V2” represents the volume of the sample extraction solution added during the sample extraction process (μL), “V3” represents the volume of the supernatant collected during the sample extraction process (μL), and “m” represents the mass of the weighed sample (g). Hierarchical cluster analysis (HCA) was conducted utilizing the R package pheatmap, employing the Euclidean distance coefficient. Partial least squares discriminant analysis (PLS-DA) was employed to identify differentially expressed metabolites (DEMs). To ensure the robustness of the PLS-DA model and mitigate overfitting, validation was performed using standard metrics, including R^2^X, R^2^Y, Q^2^, and permutation tests. To ensure consistency between the multivariate statistical framework and the VIP-based selection of differentially accumulated metabolites (DEMs), a sequential minor modification of the analytical pipeline was followed [40]. The partial least squares discriminant analysis (PLS-DA) was performed for each pairwise comparison (CK vs. each treatment group) using the mixOmics, R package (version 6.32.0). Model validity was assessed by 10-fold cross-validation and 200 permutation tests to avoid overfitting (all Q^2^ values > 0.6, permutation *p* < 0.05). The Variable Importance in Projection (VIP) score was calculated from the final validated PLS-DA model for each metabolite. Finally, metabolites with VIP > 1.0, absolute fold change (FC) > 2 or <0.5, and FDR-adjusted *p* < 0.05 (Benjamini–Hochberg correction) were defined as significant DEMs. This stepwise combination of multivariate (PLS-DA + VIP) and univariate (FC + FDR) thresholds is consistent with current best practices for plant metabolomics and avoids the common inconsistency of using VIP from an unreported or unvalidated PLS-DA model. A total of 24 DEMs were identified across nine pairwise comparisons, and KEGG enrichment analyses were performed.

### 4.5. Transcriptomic Analysis

Total RNA was extracted from YZ05-51 sugarcane leaf sheath samples using TRIzol reagent (Sangon Biotech Co., Ltd., Shanghai, China) according to the manufacturer’s instructions. RNA quality was verified using an Agilent 2100 Bioanalyzer. All samples exhibited high integrity with RNA Integrity Number (RIN) values ≥ 7.0. Ribosomal RNA (rRNA) contamination was negligible in all samples. Sequencing libraries were constructed with an average insert size of approximately 150 bp. High-quality RNA was used for library construction clustering, and the (Illumina HiSeq^TM^ 2500 platform, Illumina Inc., San Diego, CA, USA), was used for pair-end sequencing. The sequencing depth of the samples was 3 Gb; FastaQC and Trimmomatic were used for quality assessment and quality cutting, and low-quality bases (Q < 20) were removed. Comprehensive sequencing quality control showed that clean reads had Q20 ≥ 99.15%, Q30 ≥ 97.22% (with a mean Q30 > 97.38%), and GC content ranging from 55.01% to 56.38%, indicating high-quality sequencing data. A total of 10,000 sequences was extracted from the clean data and subjected to a BLASTn alignment with the NCBI NT database. The alignment results were filtered based on an E-value ≤ 1 × 10^−10^, a similarity > 90%, and coverage > 80%. Clean reads were mapped to the R570 sugarcane genome sequence using the HISAT2 v2.2.1 software [57].

Gene expression levels were estimated using transcripts per million (TPM). Fold-change (FC) between samples was calculated to assess the magnitude of expression differences between groups. Genes with significant expression changes were identified using DEGseq v1.56.1 software with a |log2Fold Change| > 2 and a q-value < 0.05. DEGseq was selected over DESeq2, and edgeR as suited the sugarcane transcriptomic dataset. Unlike these negative binomial tools optimized for diploid species with sufficient biological replicates, DEGseq uses a Poisson-based algorithm robust to the non-normal, skewed-read count distributions of polyploid sugarcane transcriptomes. For three biological replicates (n = 3) per group, DEGseq produced more stable differential expression results, whereas DESeq2 and edgeR tended to introduce overcorrection and elevated false discovery rates. Gene function enrichment analysis was performed by comparing Gene Ontology (GO) and Kyoto Encyclopedia of Genes and Genomes (KEGG) data. Fisher’s exact test was used to select significant GO categories and KEGG pathways with an FDR < 0.05. In addition, the annotated gene functions were based on the following databases: non-redundant protein sequences (NR), cluster of orthologous groups (KOG), and among the DEGs were predicted. The expression levels of key genes identified in the metabolomic and transcriptomic datasets were analyzed using quantitative RT-PCR (qRT-PCR), according to the manufacturer’s instructions (PerfectStart AQ602, Transgene Biotech, Beijing, China). Sixteen genes were selected for qRT-PCR validation, representing core functional categories from the integrated transcriptomic and metabolomic analysis. Specific primers for qRT-PCR were designed (Supplementary Data S9). Each qPCR reaction contained three biological and three technical replicates. PCR was performed using an Applied Biosystems 7500 Real-Time PCR system (Foster City, CA, USA). The *GAPDH* gene was used as the internal control for the normalization of expression, and relative expression was estimated using the delta Ct method (2^−ΔCt^).

## 5. Conclusions

This study demonstrated that YZ05-51 was the best variety for enhancing sucrose content after spraying Arg (20 g/mu) among the three varieties. It also revealed distinct differences in sucrose metabolism and how Arg regulates sucrose metabolism in YZ05-51 sugarcane under different pH conditions, generating extensive transcriptomic and metabolomic datasets. These findings highlight the complexity of sucrose metabolism and necessitate further research to fully understand the regulatory mechanisms. YZ05-51 exhibited a strong response to Arg treatment, with significant changes in DEGs and DEMs influenced by the genetic background, Arg concentration, and pH levels. Coordinated shifts in metabolite accumulation and gene expression suggest that Arg treatment correlates with upregulated expression of sucrose transport-related genes, suggesting potential enhancement of transport capacity, including starch and sucrose metabolism, glycolysis/gluconeogenesis, amino sugar metabolism, galactose metabolism, and ABC transporters. The knockout or overexpression of SBEs and TPS1 specifically in YZ05-51 sugarcane serves to validate their roles in arginine-mediated sucrose accumulation. To elucidate the roles of ABC transporter genes, which are upregulated by arginine, further functional studies should employ heterologous expression or sugarcane transgenics. Transport assays and localization studies are essential to confirm their involvement in the enhanced translocation of sucrose to the culm. Arginine may enhance the sucrose synthesis and transport capacity of leaf sheaths by increasing nitrogen nutrition and cell membrane activity. While improving sucrose transport efficiency, it is accompanied by a reduction in other sugars inside the leaf sheaths. Although sugarcane leaf sheaths are not the main site of sucrose synthesis, the increased metabolic capacity of leaf sheaths after arginine treatment promotes the exertion of transport capacity. Overall, these insights provide a foundation for optimizing nutrient management strategies in sugarcane, and further research is needed to explore DEGs and DAMs in sugar metabolism for improved agricultural practices.

## Data Availability

The datasets presented in this study can be found in online repositories. (https://ngdc.cncb.ac.cn/databasecommons/) Bio project submissions ID: subPRO094502, Accession No: PRJCA064853; accessed on 21 May 2026.

## Supplementary Materials

The following supporting information can be downloaded at: https://www.mdpi.com/article/10.3390/ijms27125476/s1.
