# Supplementary material for: Integrated Metabolomics and Transcriptomics Analysis of Exogenous Arginine-Mediated Sucrose Accumulation in Sugarcane
Source: Int J Mol Sci. 2026 Jun 17;27(12):5476. doi: 10.3390/ijms27125476 (PMC13299503; doi:10.3390/ijms27125476)
Supplement: Supplementary file 1 [file ijms-27-05476-s001.zip › All Supplementary Figure S1-S5.pptx]

## Slide 1
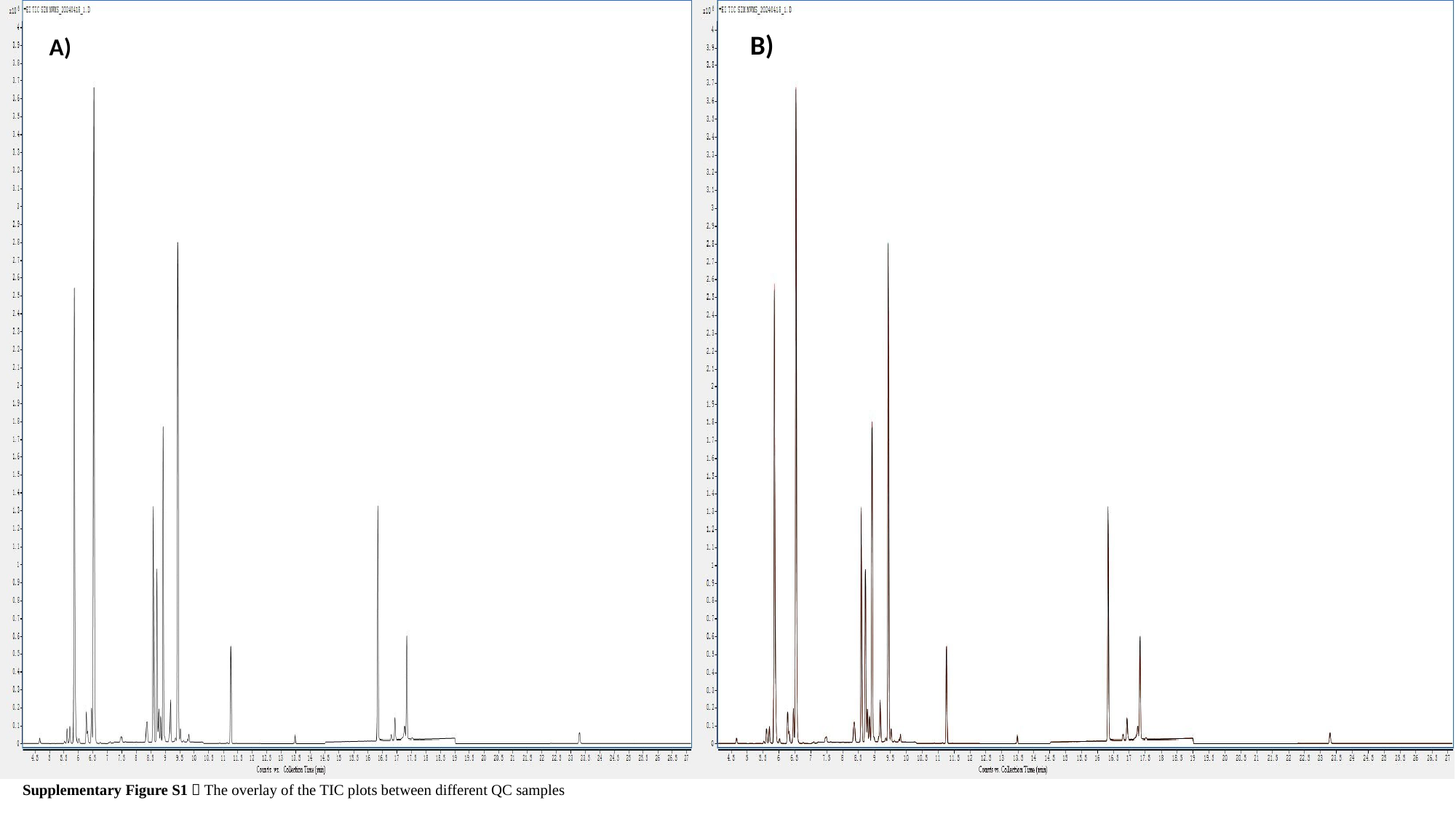

B)
A)
Supplementary Figure S1：The overlay of the TIC plots between different QC samples

## Slide 2
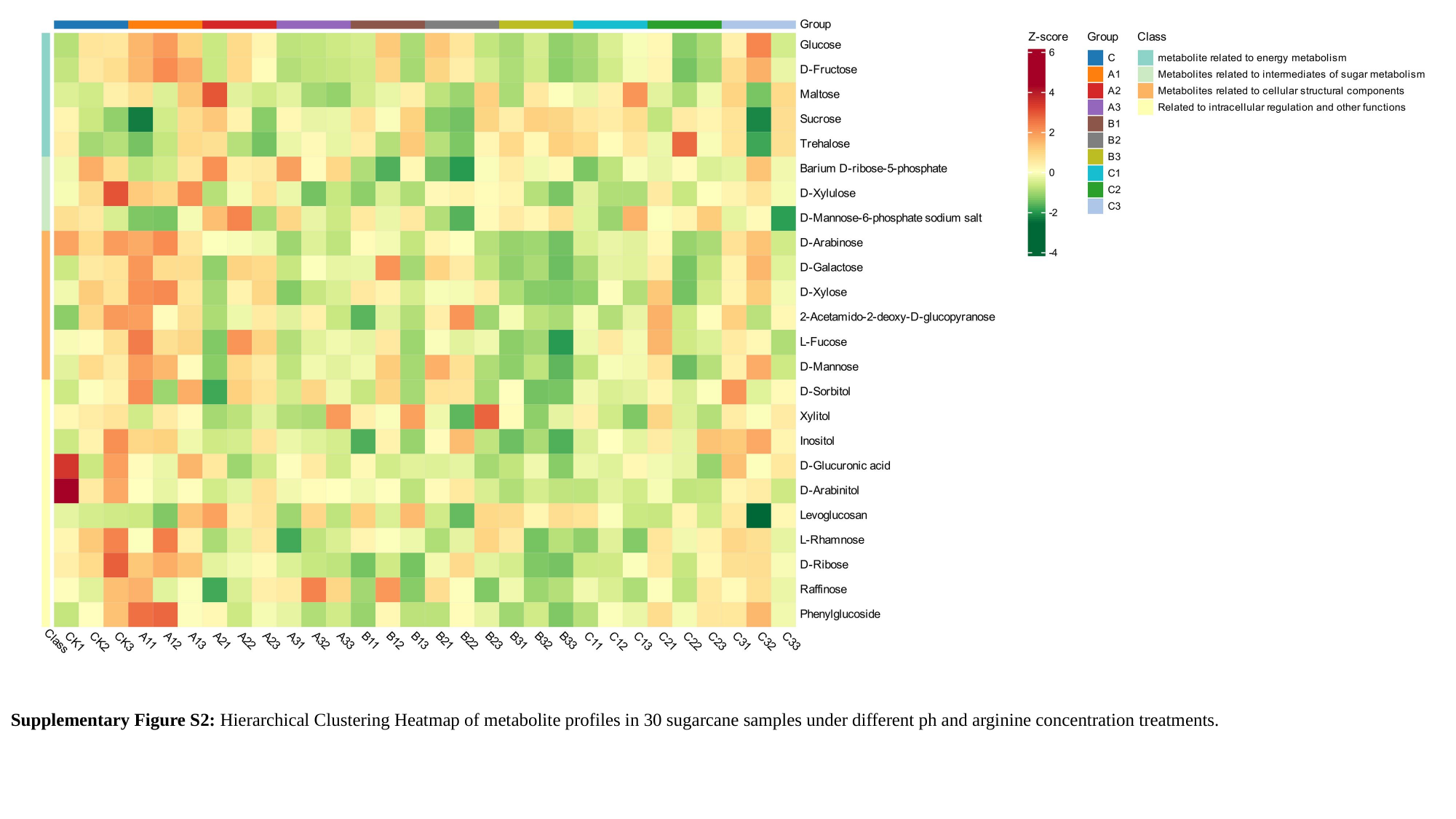

Supplementary Figure S2: Hierarchical Clustering Heatmap of metabolite profiles in 30 sugarcane samples under different ph and arginine concentration treatments.

## Slide 3
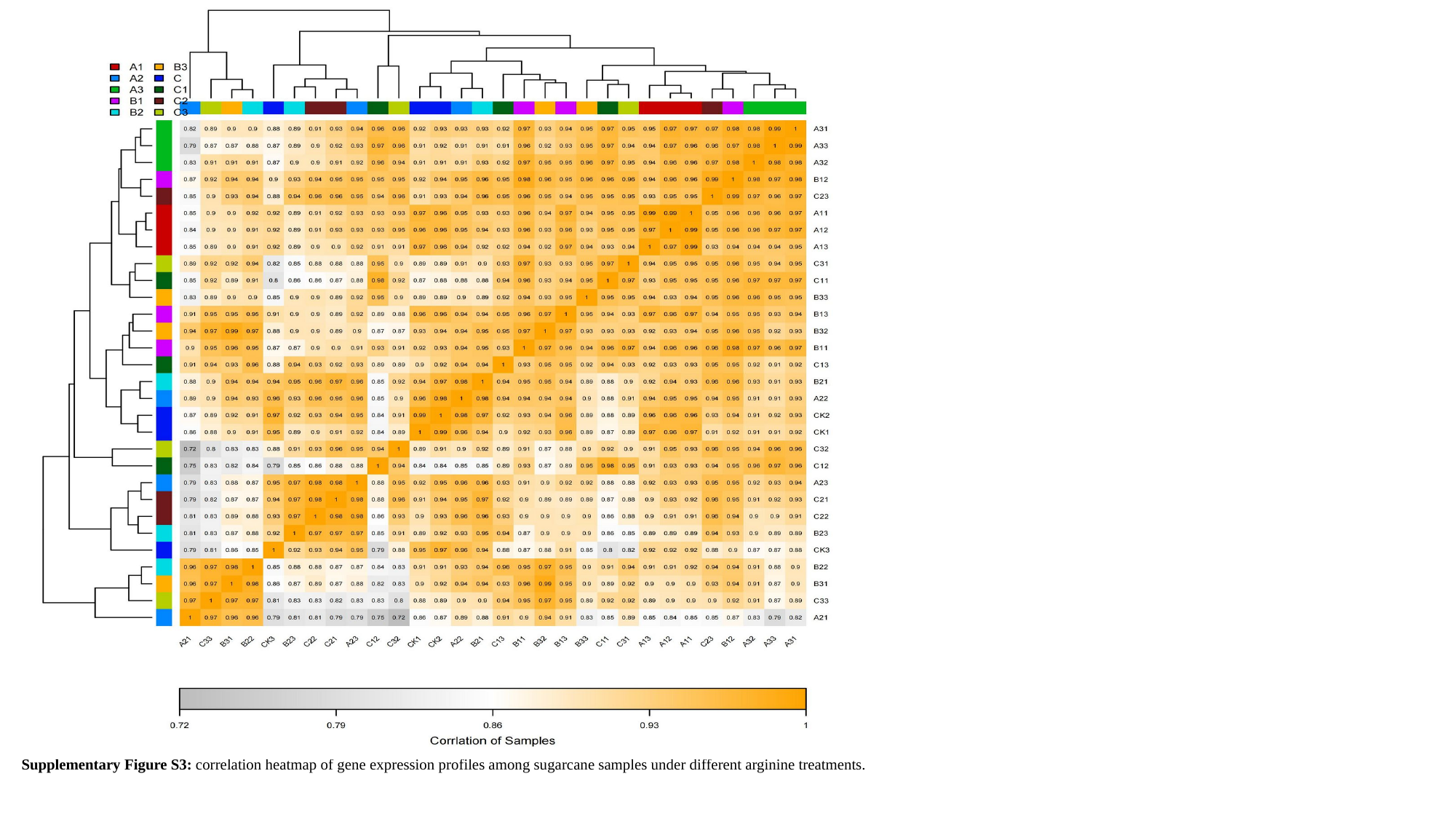

Supplementary Figure S3: correlation heatmap of gene expression profiles among sugarcane samples under different arginine treatments.

## Slide 4
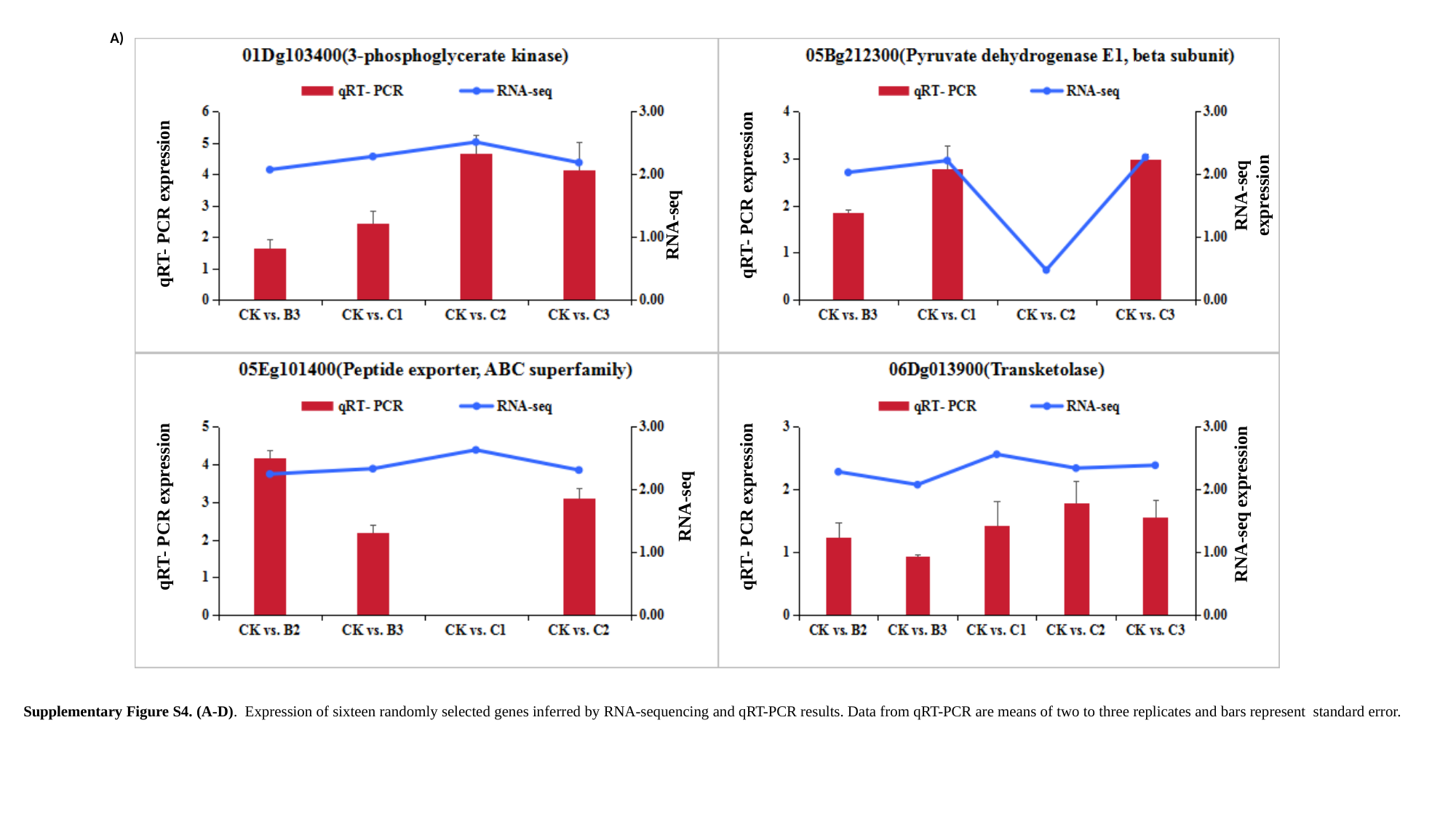

A)
RNA-seq
qRT- PCR expression
qRT- PCR expression
RNA-seq expression
RNA-seq
RNA-seq expression
qRT- PCR expression
qRT- PCR expression
Supplementary Figure S4. (A-D). Expression of sixteen randomly selected genes inferred by RNA-sequencing and qRT-PCR results. Data from qRT-PCR are means of two to three replicates and bars represent standard error.

## Slide 5
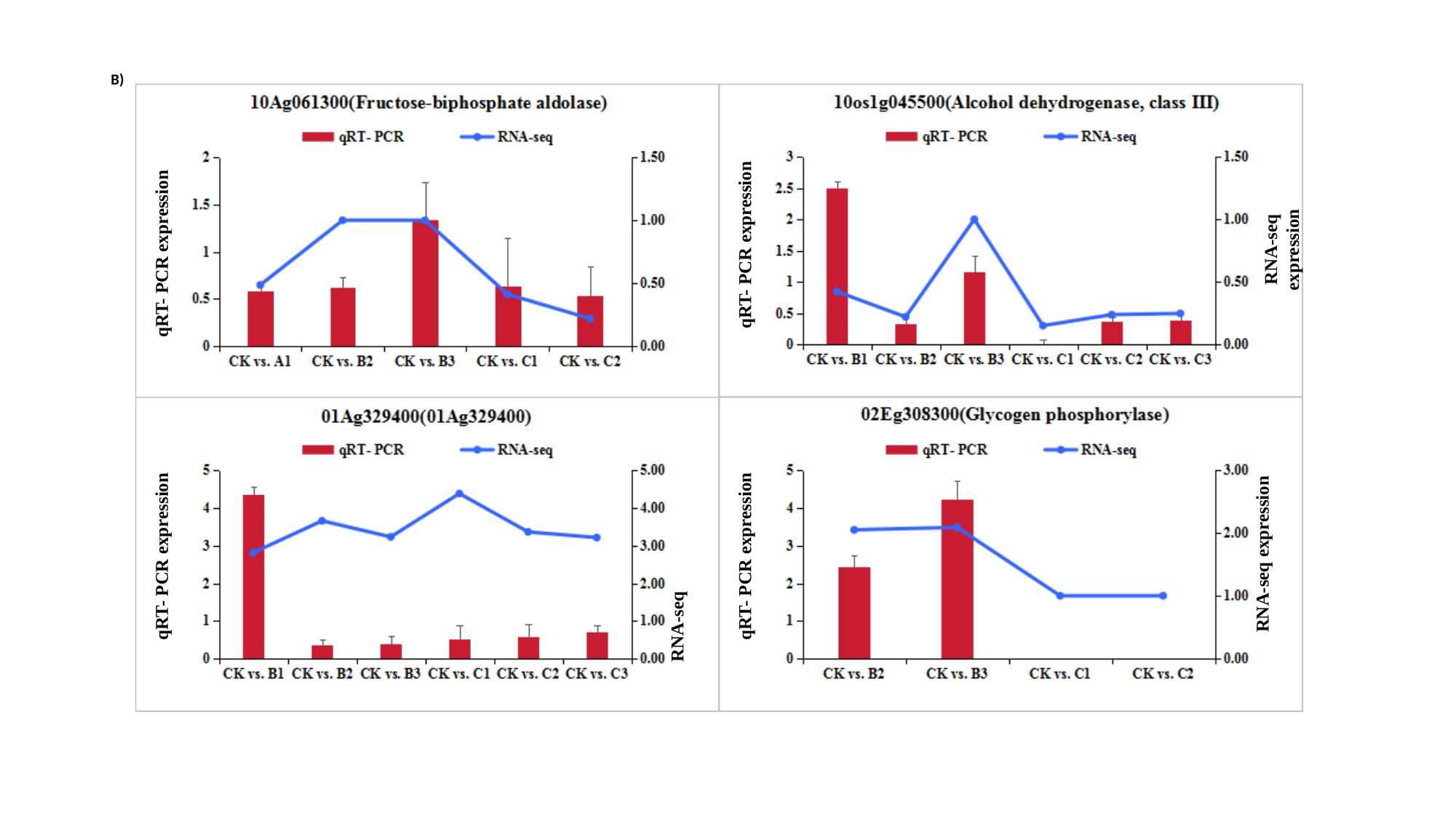

B)
qRT- PCR expression
qRT- PCR expression
RNA-seq expression
RNA-seq expression
qRT- PCR expression
qRT- PCR expression
RNA-seq

## Slide 6
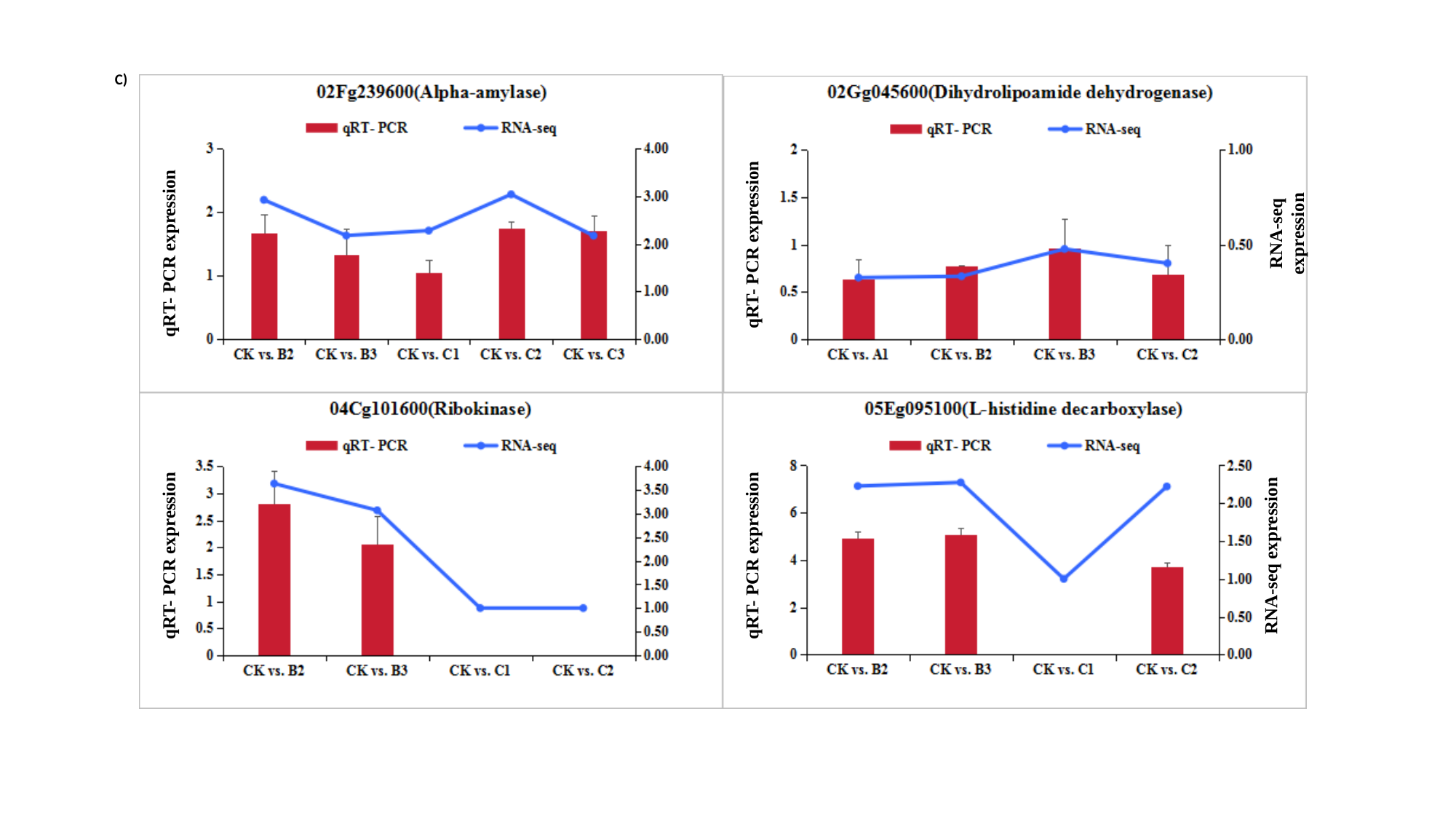

C)
qRT- PCR expression
RNA-seq expression
qRT- PCR expression
RNA-seq expression
qRT- PCR expression
qRT- PCR expression

## Slide 7
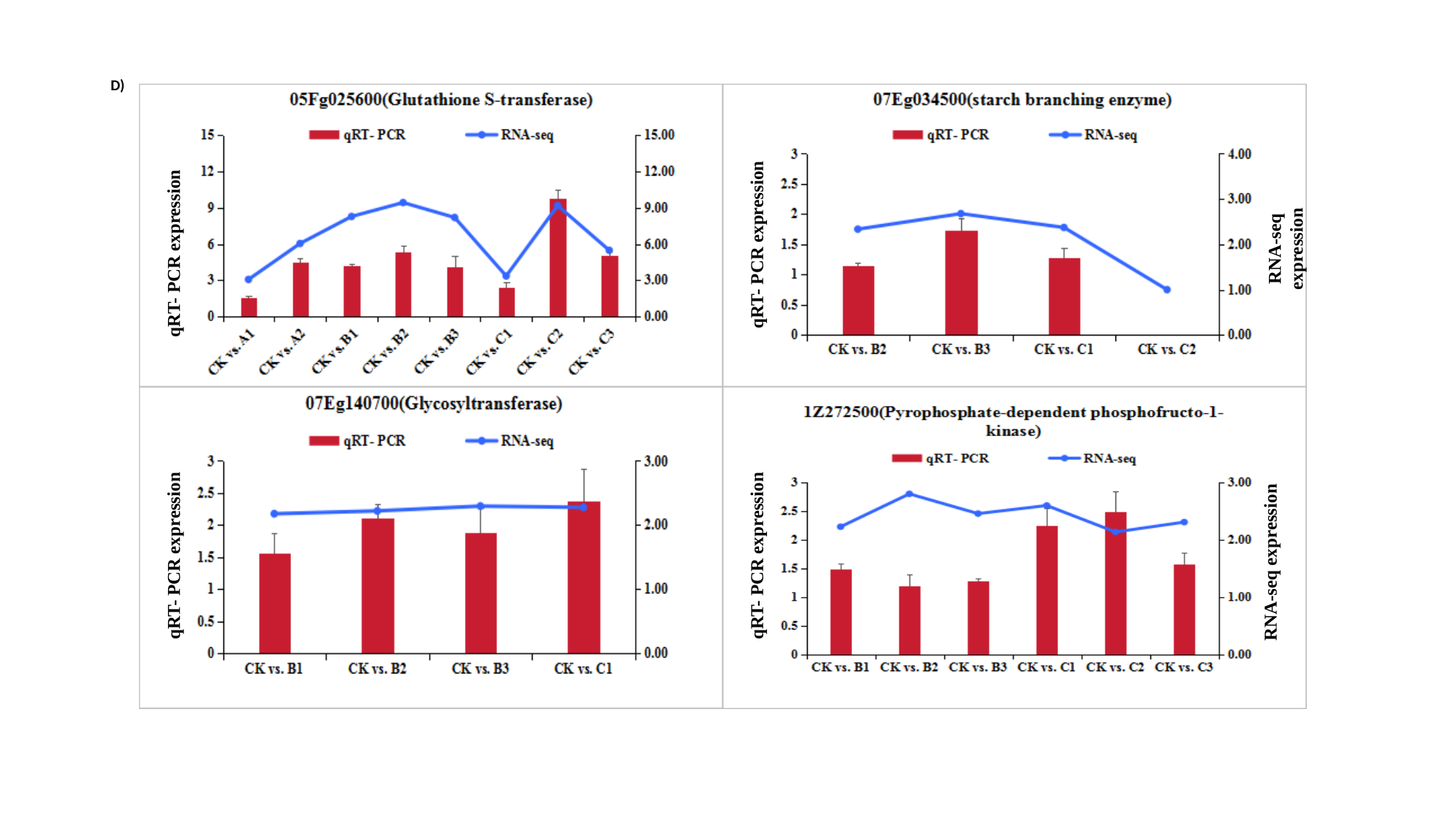

D)
qRT- PCR expression
qRT- PCR expression
RNA-seq expression
qRT- PCR expression
qRT- PCR expression
RNA-seq expression

## Slide 8
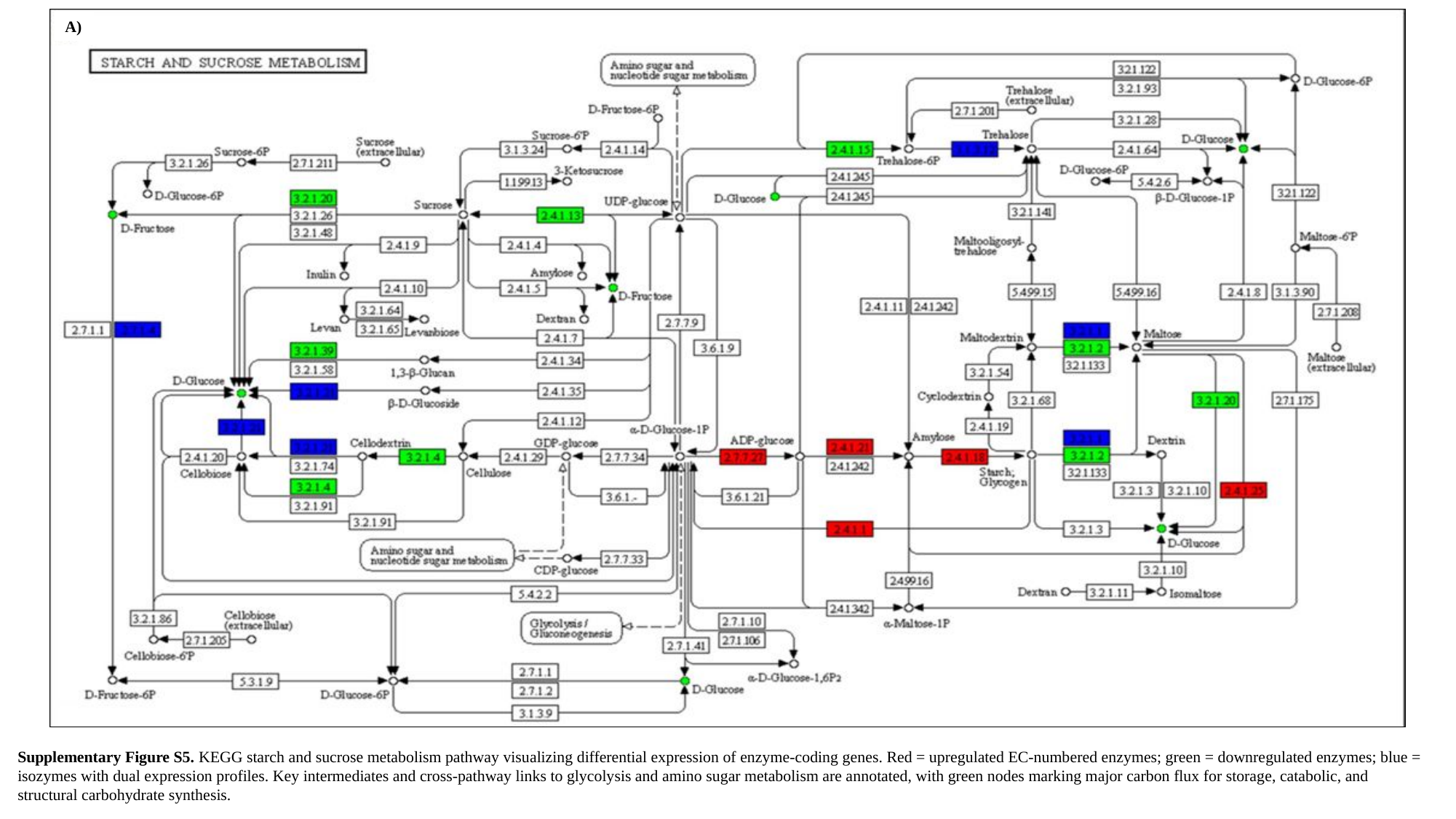

A)
Supplementary Figure S5. KEGG starch and sucrose metabolism pathway visualizing differential expression of enzyme-coding genes. Red = upregulated EC-numbered enzymes; green = downregulated enzymes; blue = isozymes with dual expression profiles. Key intermediates and cross-pathway links to glycolysis and amino sugar metabolism are annotated, with green nodes marking major carbon flux for storage, catabolic, and structural carbohydrate synthesis.

## Slide 9
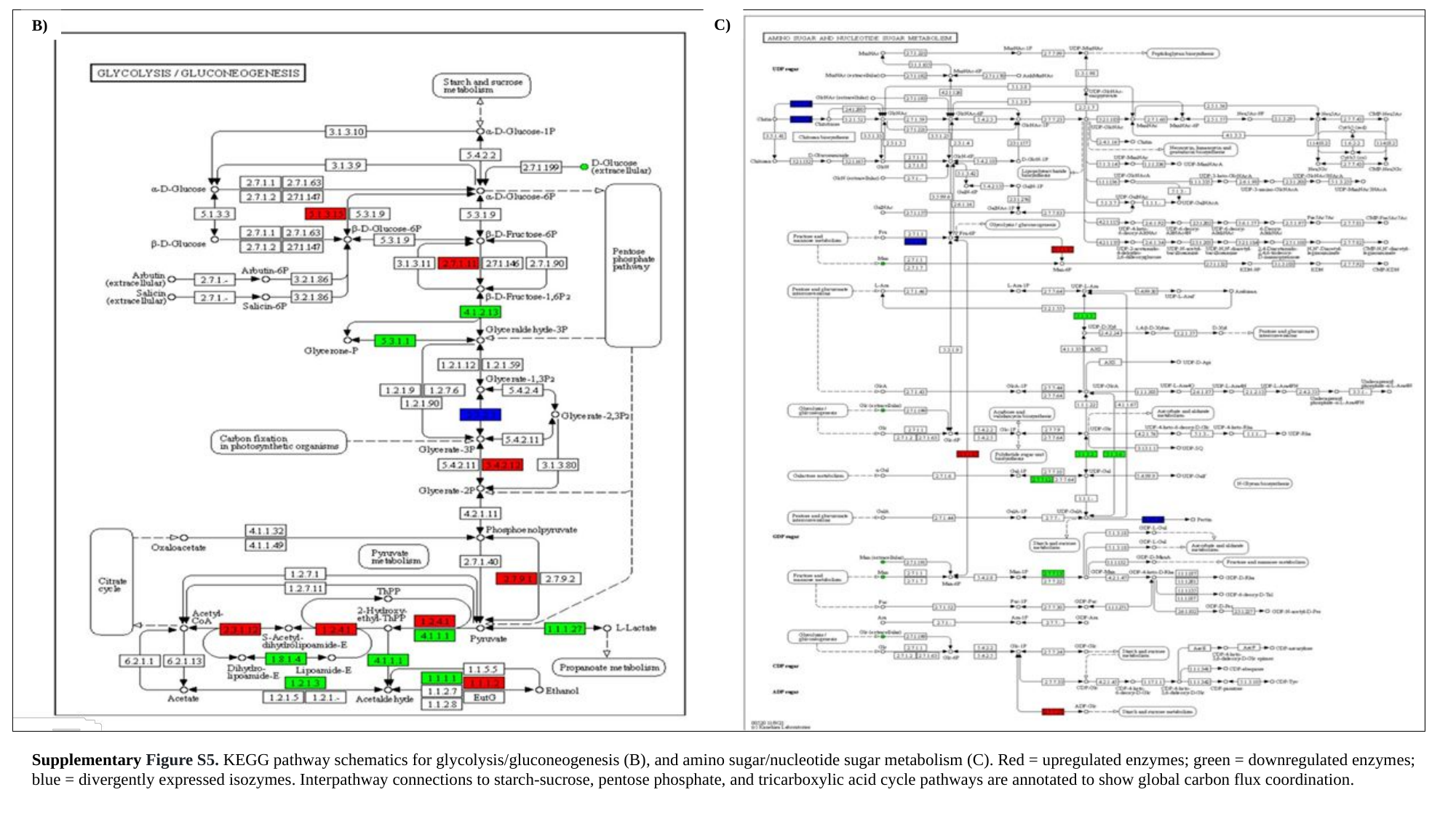

C)
B)
Supplementary Figure S5. KEGG pathway schematics for glycolysis/gluconeogenesis (B), and amino sugar/nucleotide sugar metabolism (C). Red = upregulated enzymes; green = downregulated enzymes; blue = divergently expressed isozymes. Interpathway connections to starch-sucrose, pentose phosphate, and tricarboxylic acid cycle pathways are annotated to show global carbon flux coordination.

## Slide 10
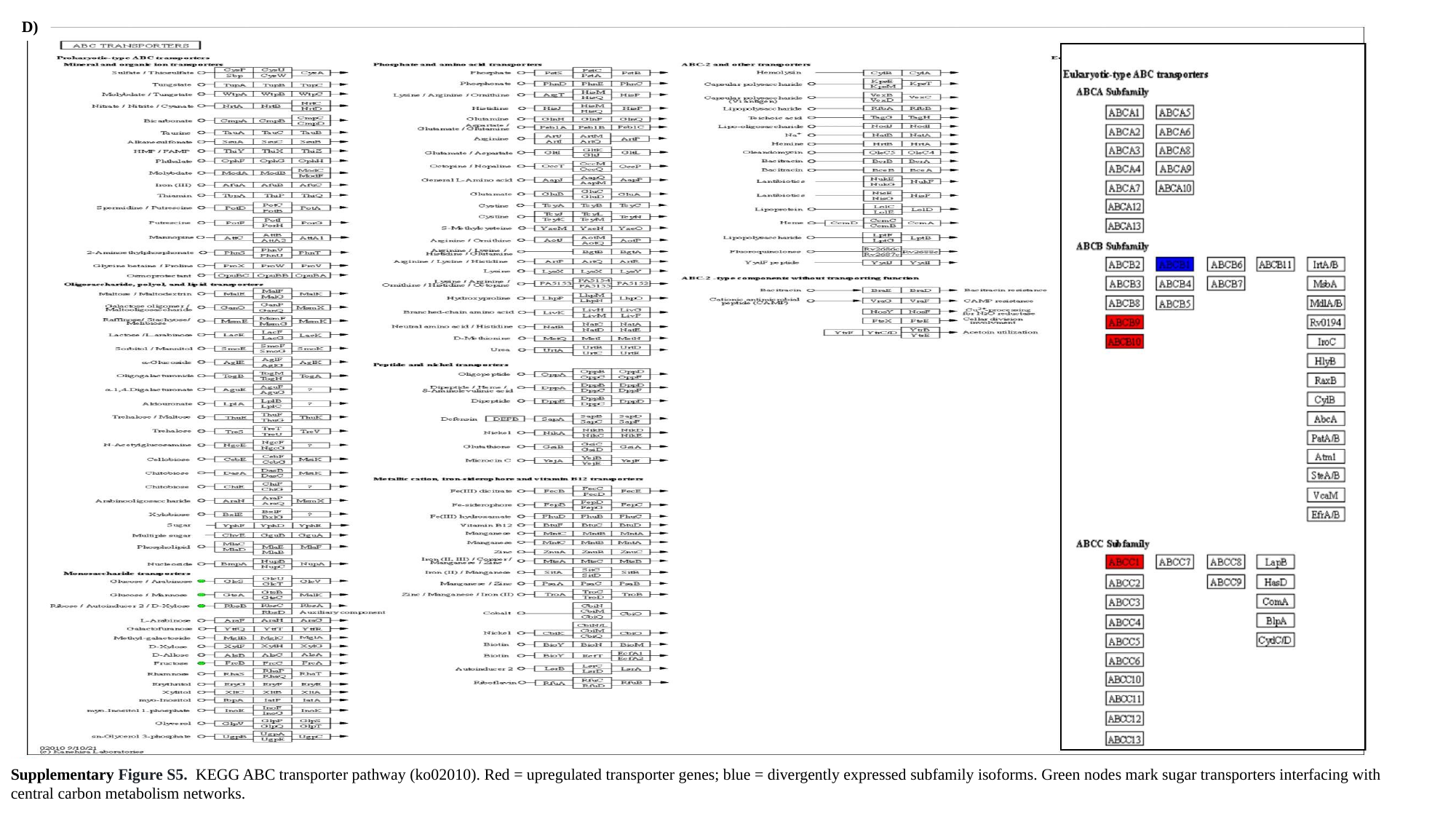

D)
Supplementary Figure S5. KEGG ABC transporter pathway (ko02010). Red = upregulated transporter genes; blue = divergently expressed subfamily isoforms. Green nodes mark sugar transporters interfacing with central carbon metabolism networks.
